# Short implants compared to regular dental implants after bone augmentation in the atrophic posterior mandible: umbrella review and meta-analysis of success outcomes

**DOI:** 10.1186/s40729-023-00476-0

**Published:** 2023-07-04

**Authors:** Gustavo Sáenz-Ravello, Benjamín Ossandón-Zúñiga, Vicente Muñoz-Meza, Dante Mora-Ferraro, Mauricio Baeza, Shengchi Fan, Keyvan Sagheb, Eik Schiegnitz, Leonardo Díaz

**Affiliations:** 1 Faculty of Dentistry, Center for Epidemiology and Surveillance of Oral Diseases, University of Chile, Santiago, Chile; 2 Faculty of Dentistry, University of Chile, Santiago, Chile; 3 Faculty of Dentistry, Postgraduate School, University of Chile, Santiago, Chile; 4 Department of Oral and Maxillofacial Surgery, University Medical Center of the Johannes-Gutenberg University, Augustusplatz 2, 55131 Mainz, Germany

**Keywords:** Short dental implants, Dental implants, Bone regeneration, Atrophic mandible, Evidence-based dentistry, Umbrella review, Meta-analysis

## Abstract

**Purpose:**

To assess the body of evidence of short versus regular implants after bone augmentation (BA) in the atrophic posterior mandible in the context of implant treatment success outcomes.

**Methods:**

Seven databases, two registries, and reference lists were searched for systematic reviews and meta-analysis (SR/MA), randomized controlled trials (RCTs) and longitudinal studies published in English, Spanish or German since 2012. Confidence in the SR/MA methodology was evaluated using AMSTAR-2 and the risk of bias of primary studies using Cochrane’s RoB 2.0 and ROBINS-I. A random-effects meta-analysis and a meta-regression were performed for continuous and dichotomous outcomes. GRADE approach was used to assess the certainty of the evidence.

**Results:**

Eighteen SRs/MAs, most of them “critically low” and “low” confidence with substantial overlap, included 14 relevant RCTs with a high risk of bias. A cohort study with moderate risk of bias was added. Quantitative synthesis of 595 implants and 281 hemiarches/patients indicates that the use of short implants (< 10 mm) compared to regular implants and BA may reduce implant failure at 1-year follow-up, and marginal bone loss (MBL) at 3-, 5-, and 8-year follow-up; is likely to reduce the risk of biological complications at 1-, 3-, 5-, and 8-year follow-up; and may be the patient's preferred alternative. There is a correlation between bone height, MBL and biological complications.

**Conclusions:**

The available evidence partially suggests that the use of short implants could decrease implant failure, MBL, and biological complications, and increase patient satisfaction. However, given the need for further RCTs and real-world evidence to fully evaluate short- and long-term outcomes, it would be prudent for clinicians to carefully consider the individual needs and circumstances of the patients before deciding whether to use short implants.

*Trial registration* PROSPERO CRD42022333526

**Graphical Abstract:**

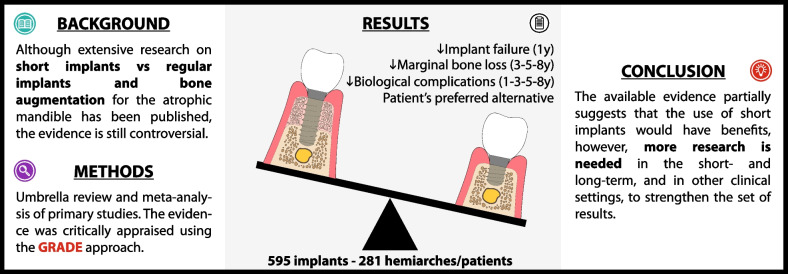

**Supplementary Information:**

The online version contains supplementary material available at 10.1186/s40729-023-00476-0.

## Introduction

The challenge of implant-supported rehabilitation of the atrophic mandible could be summarized in three points of view: the skill and expertise required for the correct performance of the surgical technique; the anatomical characteristics of the surgical site, anterior or posterior, proximity to the inferior alveolar nerve or other adjacent structures, such as the floor of the mouth (and sublingual gland) and muscle insertions (buccinator and mylohyoid), density of cortical and bony characteristics [1]; and the patient, in relation to their age and adherence to treatment [2].

Nowadays, one of the most widely accepted therapeutic options is the placement of regular, standard-length, or conventional endosseous implants over an edentulous site that has been operated to increase the available three-dimensional bone volume. However, there is a considerable complication rate in these procedures: 6.8–57.1% for distraction osteogenesis, 2.5–100% for bone blocks and 5.8–27.3% for guided bone regeneration [3, 4].

The placement of short implants on an atrophic but pristine alveolar ridge is an alternative to avoid frequent comorbidity and long recovery time compared with bone augmentation (BA) surgery. Currently, 34 systematic reviews and meta-analyses (SR/MA) from the PROSPERO database suggest that short implants may be more predictable than BA techniques [5–7]. However, extensive evidence regarding the possible advantages of these techniques is still controversial.

Furthermore, the concept of short implants is still unclear; some studies had considered < 10 mm, while others later defined it as < 8, < 7, < 6, or 4 mm [8–10]. Similarly, there is high heterogeneity in the methodology used to synthesize and communicate these results. Efforts to provide evidence for decisions in daily clinical practice are needed. In this sense, overviews or umbrella reviews (UR) use explicit and systematic methods to search for and identify multiple SR that address a health problem, to extract and analyze their results through important outcomes [11]. The purpose of this UR is to answer the question “What are the benefits or harms of using short implants (I) vs. regular implants after BA (C) in the posterior atrophic mandible (P) in the context of implant treatment success outcomes (O)?

## Materials and methods

### Protocol and study design

The protocol for this UR was registered in PROSPERO (CRD42022333526) and developed following the recommendations of the Cochrane Overview of Reviews [11], PRISMA 2020 guidelines [12], and PRIO recommendations [13].

This study was conducted in two phases. For the first, a search and identification of SR/MA was carried out, under the criteria that contained an MA, a paragraph or a table that answered the exact question of this UR. In this sense, it was categorized as “Broader” if the review addressed this question, other populations and interventions; “Exact”, if it addressed the same population and intervention; “Narrow” if it addressed a more specific aspect of the population or intervention evaluated in this UR. The second phase consisted of identifying the primary studies included in the SR/MA, observing the overlap between them, and subsequently assessing their eligibility with the PICO question of this UR. Likewise, a search for primary studies published in the last 10 years was carried out. Then, the risk of bias of the primary studies included was evaluated to finally perform a meta-analysis of the pertinent articles.

### Eligibility criteria

The focused clinical question of this UR is detailed below:

- Participants (P): patients with atrophic partial or fully edentulous posterior mandibular ridge.
- Intervention (I): short implants (< 10 mm) placed on the native bone.
- Comparison (C): regular length implants (≥ 10 mm) placed on previously augmented bone by distraction osteogenesis, inlay/onlay bone block graft or guided bone regeneration (GBR).
- Implant treatment (O) success outcomes [14]:
  - Primary: (1) implant survival/failure; (2) marginal bone loss (MBL); (3) biological complications.
  - Secondary: (4) prosthetic failure or complications, (5) patient-reported outcome measures, and (6) costs or other economic analysis.
- Study types: SR/MA (according to the definition provided by the “Cochrane Handbook for Systematic Reviews of Interventions”, Version 6.3, 2022) of randomized clinical trials (RCTs) or longitudinal studies were included in the first phase. For the second-phase RCTs published after the last search for the most recent SR/MA, and non-randomized clinical trials or longitudinal studies reported within the last 10 years were included. In this regard, the expected low certainty on the evidence was supplemented with the inclusion of longitudinal cohort studies of two or more intervention arms (prospective and retrospective) [15].
- Restrictions on study selection: articles and conference abstracts published or in press from the last 10 years (2012–2022), available in English, Spanish, or German (languages in which the authors of this review are proficient). The last 10 years were considered a restriction, because most SR/MA were published during this period.
- Exclusion criteria:
  - P: surgical techniques performed exclusively on the maxilla.
  - I and C: studies comparing short implants with each other or comparing two implant systems both placed in the native bone.

### Information sources and selection process

The literature search was based on PRESS recommendations [16] and was performed independently by four reviewers (GSR, BOZ, DMF, and VM) in the Cochrane Library, ClinicalTrials.gov, EBSCOhost (Dentistry & Oral Sciences Source), LiLACS, PubMED, SciELO, Scopus, and Web of Science. The algorithms used to conduct the search SR/MA, RCTs and cohort studies were developed by an experienced reviewer (GSR) and an experienced oral surgeon (LD), starting from the PubMED thesaurus (MeSH terms) that were adapted to the other platforms (Additional file 1: Table S1).

Studies were added to the Rayyan platform (https://www.rayyan.ai/) to eliminate duplicates. They were screened by four reviewers (GSR, BOZ, DMF, and VM) by independent assessment of title–abstract–keywords compared to the inclusion criteria. A Fleiss test was computed in Microsoft Excel 2022 (Microsoft Corporation, Redmond, USA) to assess inter-rater agreement for more than two reviewers. Interrater agreement was interpreted according to the categories proposed by Landis and Koch [17]. From this screening, studies compatible with full-text reading were independently selected (GSR, BOZ, DMF, and VM). Likewise, through full-text reading, other SR/MA, RCTs and cohort studies cited and included in the reference list were identified, using the same terms for the search in the databases and registries, and their eligibility was discussed. Authors were contacted via e-mail to request key information that was not reported in the included studies.

### Data collection process

One of the limitations reported regarding UR is the overlapping of primary studies [18], which decreases the precision obtained in the performed synthesis. To manage this, tools provided by Epistemonikos.org were used, automatically detecting, based on artificial intelligence, the primary studies belonging to each of the SR, generating as a result an “Evidence Matrix”, from which the overlapping was calculated using the Corrected Coverage Area Index (CCA) [19]. Based on this matrix, each of the four reviewers checked the primary studies against the PICO question of this review, and then independently extracted their data (GS, BO, VM and DM), which was subsequently verified by a peer reviewer. Based on the table “Characteristics of included studies” provided by the Cochrane Collaboration [20], using a Microsoft Excel 2022 (Microsoft Corporation, Redmond, USA) spreadsheet, the following items were extracted:

- For SR:
  - Number and date of the last search, PICO (and reported effects), number, design of included studies and relation to the PICO question of the present UR (scope).
- For primary studies:
  - Methodological study design, follow-up time, setting, and country.
  - Patient characteristics: number of participants and implants, age/sex, mandible area (premolar–molar)/type of edentulism (partial or complete), bone characteristics (height, width, Cawood classification, other classification).
  - Surgical protocol: antibiotic prophylaxis, implant system and length/diameter, raised flap (Y/N), insertion torque, and bone augmentation approach.
  - Prosthesis parameters: loading protocol (provisional to definitive loading; immediate, early, conventional), type of prosthesis, retention method (cemented/screw)/implant–abutment connection, splinted (Y/N), crown/implant ratio.
  - Risk factor assessment: Bruxism, smoking, history of periodontitis/maintenance time, and local infection (before and/or after surgery).
  - Costs.

### Confidence and risk of bias assessment

A panel composed of three reviewers assessed the confidence for SR/MA and the risk of bias of primary studies (GS, MB, and LD). Regarding the confidence of the SRs, the AMSTAR-2 guideline 16 items [21] was used according to the identification of weaknesses in critical aspects (items 2, 4, 7, 9, 11, 13, and 15). Subsequently, confidence was categorized as critically low, low, moderate or high. Discrepancies were resolved through consensus. The risk of bias of the primary studies was obtained from the assessments made by the authors of the best SRs included. If any discrepancies were present, a risk of bias assessment of the primary study was performed using the Cochrane risk-of-bias tool for randomized trials (RoB 2) [22] or ROBINS-I tool [23]. The use of the tools described above allows for a better comparison of evidence from RCTs and non-randomized studies, because they sit on a common risk of bias metric [24].

### Data synthesis and effect measures

Cochrane Collaboration’s Review Manager 5.4.1 was used to perform a meta-analysis of the primary studies. If those studies did not qualify for quantitative synthesis (< 2 studies for the outcome assessed), a narrative summary of their results was performed. For dichotomous outcomes, a random-effects model was run using the Mantel–Haenszel approach (if applicable, with continuity correction for “zero events” studies), generating a relative effect expressed as a Risk Ratio (RR) and its 95% confidence interval (CI). For continuous outcomes, a random-effects model was run under inverse variance, expressed as the post-intervention mean difference (MD) (95% CI). The use of the random-effects model is based primarily on definitional differences for short implants, as well as comparators reported in the literature (e.g., differences in BA techniques), estimating different, but related, intervention effects (DerSimonian and Laird approach). In addition, a trial sequential analysis (TSA) was calculated for each meta-analytic model, using a conventional boundary at *α* = 0.05 (*z* = 1.96), and an “Optimal Information Size” calculation *α* = 0.05 and *β* = 0.20 for the estimated or minimal clinically important difference in the relative effect, using alpha-spending boundary adjusted for heterogeneity. All the above calculations were performed with the software TSA 0.9.5.10 Beta (http://www.ctu.dk/tsa/downloads.aspx). The statistical units of analysis were the patient (MBL and Prosthesis failures) and the implant/hemiarch (implant failure and biological complications). To evaluate the existence of heterogeneity and the total proportion of variability due to between-study heterogeneity, chi-squared (*p* < 0.1) and *I*^2^ tests were used, respectively. If significant heterogeneity was found, a sensitivity analysis was performed to determine the impact of the inclusion or exclusion of studies (differences in the risk of bias and methodological design).

A subgroup analysis was performed according to follow-up period, bone regeneration procedure, short dental implant length (< 10, < 8, < 6, and < 4 mm) and/or diameter (if applicable). Outcomes were presented according to the analysis by subgroup or as a measure of total effect (*p* < 0.1 and *p* > 0.1 from the test for subgroup differences, respectively). In addition, a mixed-effects meta-regression using the Hartung–Knapp method for random-effects meta-analysis [25] was performed to assess the approximate mandibular bone height [4] as an independent variable for the primary outcomes (*p* < 0.05). Publication bias was investigated for each outcome by visual inspection of the asymmetries in the funnel plot, and if possible, a statistical assessment of publication bias was performed using Egger’s or Peters’ test for funnel plot symmetry (*p* < 0.05). Statistical analysis was performed using the “meta” package in R version 4.2.0 (http://www.r-project.org/index.html).

### Certainty of the evidence

First, each outcome was categorized according to its importance, as discussed by the authors of this review, based on the implant success criteria of the Harvard group, that is, at the implant level, peri-implant soft tissue level, prosthetic level, and patient satisfaction. All of the included outcomes have the same importance [14]. The certainty of the evidence for each outcome was assessed by a panel composed of the authors of this review using the GRADE guidelines with a minimally contextualized approach [26] and assessing both confidence interval and optimal information size (OIS) to facilitate the interpretation of the results from a clinical/decisional and research perspective, pondering the absolute and relative effects for the communication of the results. GRADE considers the risk of bias, inconsistency of results, indirectness of evidence, imprecision, and publication bias of each outcome. Initially, results reported from RCTs were categorized as high, and those reported from observational studies were categorized as having low certainty of evidence, with the potential to be improved (large effect, dose–response gradient, or plausible confounding effect) or downgraded. As the NRSI were assessed with ROBINS-I, the overall estimation evidence started out as high. A summary table of the results was created using the web-based software GRADEpro GDT (https://www.gradepro.org/) to present the key messages raised from this synthesis for each outcome, expressed as relative or absolute effects (if appropriate).

## Results

### Study selection

**Fig. 1:**
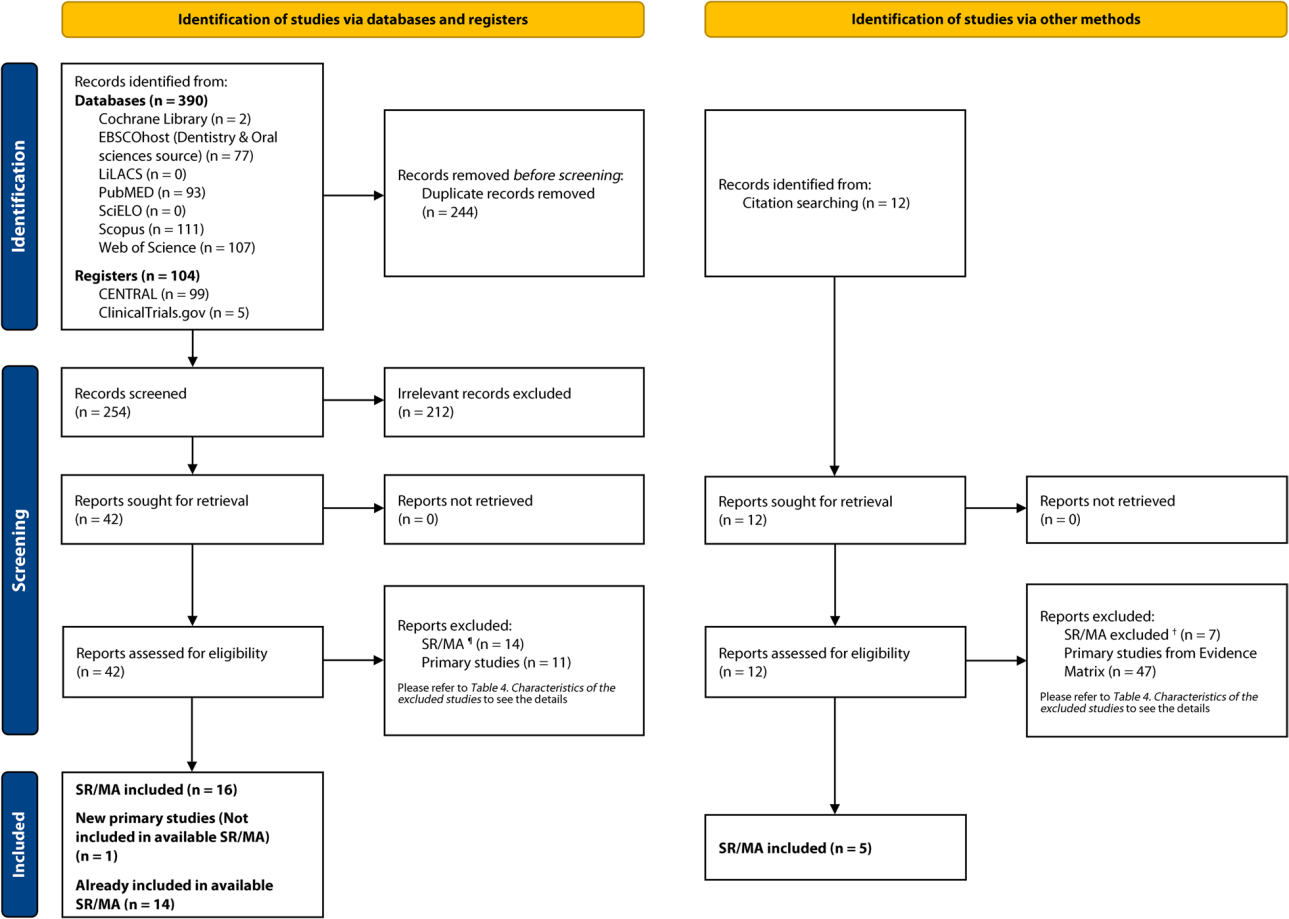
PRISMA 2020 flow chart. The number of records identified during the initial search represents the sum of all papers collected through each electronic database

The last search of all databases was conducted on June 29, 2022. A total of 494 articles were retrieved through database and registry searching, and 12 through gray literature. After removing duplicates (*n* = 244), 254 articles were screened by title–abstract–keyword reading, leaving 42 reports eligible (substantial agreement, *κ* = 0.785, 95% CI [0.733, 0.838]). After full-text reading and subsequent searching for relevant citations, 21 SR/MA were included in the first phase of this UR (93% agreement) [10, 27-46]. Subsequently, 14 primary studies [47-60] from the SR/MA included in the first phase, and 1 new primary study [61] that fit the targeted question of this UR were identified (Fig. 1) and were used for the synthesis performed in the second phase of the present review. The reasons and sources of exclusion of 79 articles are detailed in Additional file 1: Table S2.

### Characteristics of the included studies

The 21 SR/MA included (Table 1) [10, 27-46] were searched in: PubMED/Medline (100%), Cochrane Library (52%), EMBASE (52%), LiLACS (19%), Web of Science (19%), Scopus (14%), EBSCOhost (10%), Ovid (10%), ProQuest (5%), Scirus (5%), and SpringerLink (5%), and yielded a date range from 1950 to November 2020. Likewise, the use of CENTRAL (33%), ClinicalTrials.gov (5%), manual searches in specialized journals (62%), and reference lists for relevant citations (71%) were the preferred sources of gray literature. In terms of population, most studies reported mixed results in the maxilla and mandible; only seven studies [30-33, 39, 40, 43] reported the results of a primary analysis performed in the mandible. The definition of short implant ranged from 4 to 8 mm [29], 5 to 8 mm [28, 33, 42], ≤ 6 mm [31, 38, 46], ≤ 7 mm [40], less [44, 45] or ≤ 8 mm [10, 30, 32, 36, 39, 41], 8.5 mm [34] and < 10 mm [35, 37, 43].

**Table 1:**
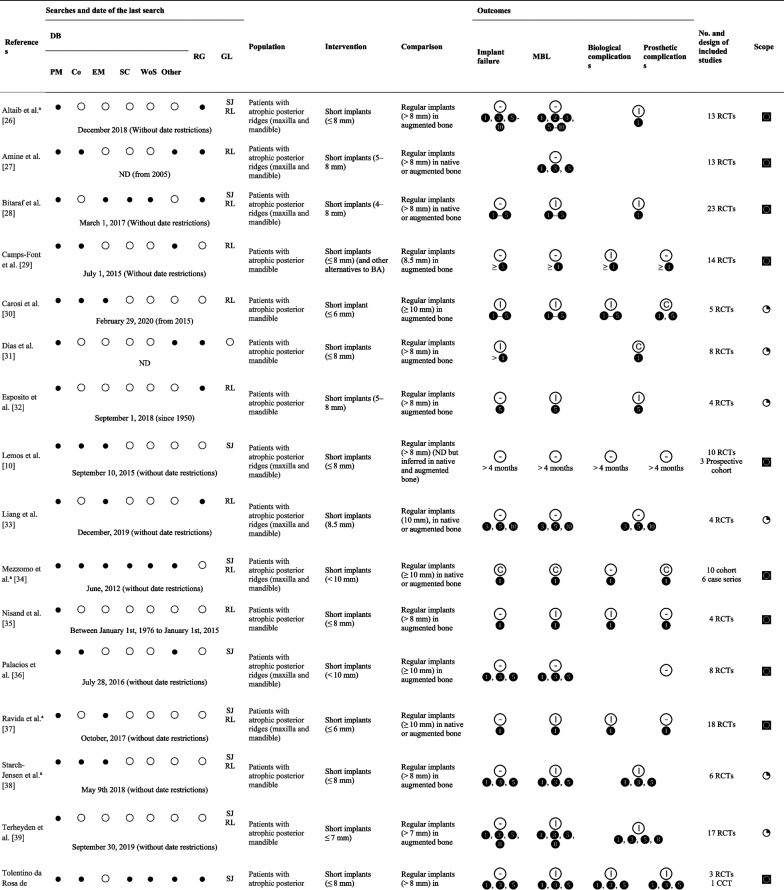

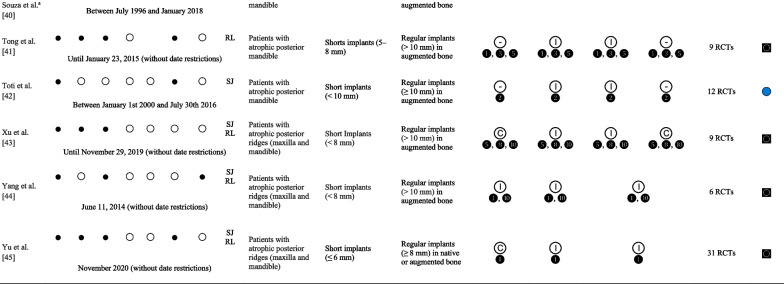
Characteristics of the included systematic review and meta-analysis

*Co* Cochrane Library, *DB* databases, *EM* EMBASE, *GL* gray literature, *PM* PubMED/Medline, *RG* registers, *SC* Scopus, *Wo* Web of Science

favors intervention;  favors comparison;  no differences between groups; ◙ broader,  exact, ◔ narrow

^a^Gray literature, ❶, ❷, ❸, ❹, ❺, ❽, ❿ years of follow-up

Thirty-eight percent of the SRs/MA included a subgroup and/or meta-regression analysis, including determinants such as age, sex, risk factors such as systemic diseases, bruxism, periodontal disease, smokers [35, 38, 46], and maxilla vs. mandible [29, 34, 35]; implant-related aspects such as length [29, 35], diameter (narrow/regular vs. wide), surface (rough vs. machined) [35], native bone vs. BA [29], different bone regeneration techniques [43], and surgical approach (one vs. two stages) [35] were evaluated. At the prosthetic level, the prosthesis type (fixed vs. removable) [34], retention type (screw vs. cemented) [35, 38], implant abutment connection type (external vs. internal) [35], and loading protocol (immediate/early vs. conventional) [35, 38, 46] were evaluated. The impact of methodological aspects such as the level of included studies (high, moderate, weak) [29], statistical approach (i.e., Bayesian meta-analysis) [32], and follow-up [34, 35] were also evaluated. One study performed a meta-regression for the mean differences in marginal bone loss (mm) between short implants and standard implants in augmented bone during the follow-up post-loading (months) with a random-effects model [37].

Most of the included SR/MA addressed a “Broader” review objective [10, 27–30, 35-38, 41, 42, 44–46] when compared to the present UR; this is in contrast to the “Narrow” [31–34, 39, 40] or “Exact” [43] relationship of the remaining SR/MA with the current UR objective. The primary studies obtained from the identified SRs/MAs presented a CCA of 31.62%. When the references are grouped by threads of studies evaluating the same cohort, the overlap increases to a CCA of 72% (“Evidence matrix” available in http://www.epistemonikos.org/matrixes/62abb8ce7aaac80c138184da).

The studies included in this review (Table 2) were split-mouth RCTs, four publication threads [48-60], two single studies, one split-mouth RCT by matrix [47], and one retrospective cohort study by search [61]. All primary studies were conducted in Italy in a private dental practice setting. Follow-up ranged from 1 to 8 years, one with a 1-year follow-up [47], four 5-year studies [48-56, 61], and one up to 8 years [57-60]. They included an initial cohort of 281 hemiarches and patients aged 37–83 years (142 hemiarches and patients for intervention and 139 for comparison). Several studies included patients who were smokers, mostly moderate; only one thread reported a heavy smoker among its participants [57-60]; at the same time, Pieri et al. [61] reported 5 patients with bruxism and 10 patients with a history of periodontitis. In the intervention group, short implants from 5 to 6.6 mm, with a diameter of 4–6 mm, were used compared to long implants from 9 to 15 mm with a diameter of 4–5 mm plus bone grafting, such as bone block alone or combined with osteogenic distraction [47]. The anatomical sites where the implants were placed were posterior and partially edentulous, with a minimum height of 5–9 mm. Most studies used antibiotic prophylaxis in the comparison group, although a minority also prescribed it for intervention [47, 61]. At surgery, all studies reported that they raised flap, with a graft healing time of 4–5 months. In total, 595 implants (291 short and 304 regular) were placed. The insertion torque was reported to be at least 25 cm in all the studies. The type of implant–abutment connection varied among the studies, between internal [47-50, 54-56, 61] and external [51-53, 57-60], and hex connection. The provisional loading time was at 4 months, and the definitive rehabilitation at 8 months, Pieri et al. [61] reported definitive loading between 4 and 5 months. The type of rehabilitation varied between studies: metal–ceramic, metal–resin or zirconia [48-56], metal–ceramic [57-60] and titanium–resin composite or zirconia–ceramic [61]. The retention method was reported indistinctly as screw-retained or cemented [48-61], or exclusively cemented [47]. With the exception of Bernardi et al. [47], who did not report the maintenance period, the majority reported a maintenance period of 4 months [48-60], up to 6 months [61].

**Table 2:** Characteristics of the primary studies included

References	Study characteristics		Group	Patient characteristics				Surgical protocol				Prosthesis parameters		
	Study design/country/setting	Follow-up		No. of participants/no. of implants	Age/gender	Mandible area/type edentulism	Bone characteristics (height/width)	Implant system	Implant length/implant diameter	Augmentation method		Loading time	Retention method	Implant–abutment connection
										Type of graft	Time to implant placement			
Pistilli et al. [48]; Felice et al. [49, 50]	RCT-SM/Italy/private dental clinic	5Y	Short	15/26	56 (37–69)/4M, 11F	Molar and premolar/partial/posterior	6–8 mm/> 5 mm	Southern Implants®, roughened grit-blasted surface (Sinergy™)	6 mm/4 mm			4M temporary/8M final	Screwed or cemented	Internal, hexagonal
			Control	15/30	56 (37–69)/4M, 11F	Molar and premolar/partial/posterior	6–8 mm/> 5 mm	Southern Implants®, roughened grit-blasted surface (Sinergy ™)	11.5, 13, 15 mm/4 mm	Interpositional block of Collagenated cancellous equine bone (OsteoBiol® Sp-Block, Tecnoss®)	4M	4M temporary/8M final	Screwed or cemented	Internal, hexagonal
Pistilli et al. [51], Gastaldi et al. [52], Esposito et al. [53]	RCT-SM/Italy/private dental clinic	5Y	Short	20/32	58.6 (39–80)/3M, 17F	Molar and premolar/partial/posterior	5–7 mm/> 6 mm	ExFeel, MegaGen Implant®, nano-structured calcium–incorporated titanium surface (Xpeed®) sanded with hydroxyapatite particles	5 mm/5 mm			4M temporary/8m final	Screwed or cemented	External, hexagonal
			Control	20/31	52.8 (42–70)/4M, 11F	Molar and premolar/partial/posterior	5–7 mm/> 6 mm	ExFeel, MegaGen Implant®, nano-structured calcium-incorporated titanium surface (Xpeed®) sanded with hydroxyapatite particles	10, 11.5, 13, 15 mm/5 mm	Interpositional block of collagenated cancellous bovine bone (OsteoBiol® Sp-Block, Tecnoss®)	4M	4M temporary/8m final	Screwed or cemented	External, hexagonal
Esposito et al. [54, 55]; Felice et al. [56]	RCT-SM/Italy/private dental clinic	5Y	Short	15/30	58.6 (39–80)/–	Molar and premolar/partial/posterior	5–7 mm/> 8 mm	ExFeel, MegaGen Implant®, nano-structured calcium-incorporated titanium surface (Xpeed®) sanded with hydroxyapatite particles	5 mm/6 mm			4M temporary/8m final	Screwed or cemented	Internal, hexagonal
			Control	15/26	52.8 (42–70)/–	Molar and premolar/partial/posterior	5–7 mm/> 8 mm	ExFeel, MegaGen Implant®, nano-structured calcium-incorporated titanium surface (Xpeed®) sanded with hydroxyapatite particles	10, 11.5, 13 mm/4 mm	Interpositional block of inorganic bovine bone (Bio-Oss®)	4M	4M temporary/8m final	Screwed or cemented	Internal, hexagonal
Felice et al. [57], Esposito et al. [58]; Felice et al. [59, 60]	RCT-SM/Italy/private dental clinic	8Y	Short	25/60	56 (40–83)/7M, 23F	–/Partial/posterior	7–8 mm/> 5.5 mm	Nanotite parallel-walled Zimmer-Biomet dental implants®, –	6.6 mm/4 mm			4M temporary/8m final	Screwed or cemented	External, hexagonal
			Control	23/61	55 (43–67)/15M, 15F	–/Partial/posterior	7–8 mm/> 5.5 mm	Nanotite parallel-walled Zimmer-Biomet dental implants®, –	9.6, 11.1, 12.6, 14.6 mm/4 mm	Interpositional block of inorganic bovine bone, particulated (Bio-Oss®)	4M	4M temporary/8m final	Screwed or cemented	External, hexagonal
Bernardi et al. [47]	RCT-SM/Italy/private dental clinic	1Y	Short	36/86	43–77/–	–/Partial/posterior	< 9 mm/–	IM Macon, Maco DentalCare®	6 mm/4.10 mm			–	Cemented	Internal, hexagonal
			Control	36/84	43–77/–	–/Partial/posterior	< 9 mm/–	ConicalActive, Maco DentalCare®	10 mm/3.90 mm	Sandwich technique, osteogenic distraction + collagenated cancellous equine bone block (Tecnoss®)	6M	–	Cemented	Internal, hexagonal
Pieri et al. [61]	Cohort study/Italy/private dental clinic	5Y	Short	23/46	57.69 ± 7.93/–	–/Partial/posterior	9–7 mm/–	OsseoSpeed™, Denstply Implants®	6 mm/4 mm			4–5M final	Screwed or cemented	Internal, hexagonal
			Control	22/51	56.4 ± 8.25/–	–/Partial/posterior	9–7 mm/–	AstraTechOsseoSpeed™, Denstply Implants®	9, 11 mm or more/3.5, 4 mm	Interpositional block of autograft + inorganic bovine bone particles Bio-Oss®)	4–5M	4–5M final	Screwed or cemented	Internal, hexagonal

### Confidence assessment and risk of bias

The distribution of certainty in the evidence was “Critically low” (33.5%), “Low” (28.5%), “Moderate” (28.5%) and “High” (9.5%) (Table 3). Within the critical items, most SR/MA established the methodology prior to conducting the study and used (item 4) adequate meta-analytic methods (according to their proposed methodology) (item 11); three studies [33, 36, 40] did not perform an adequate literature search (i.e., search at least two databases) (item 4); four studies [34, 37, 42, 43] did not list and justify the reasons for excluding primary studies (item 7); only one study [39] did not use a satisfactory technique for assessing the risk of bias (RoB) in individual studies that were included in the review (item 9); three studies [32, 34, 38] did not account for RoB in individual studies when interpreting/discussing the results of the review (item 13); finally, ten studies [10, 27, 32-34, 37, 40-43] that performed MA did not present graphical or statistical evidence for publication bias, as well as discussion of the likelihood and magnitude of its impact (item 15). Regarding noncritical items, problems were identified in relation to 1 [42], 3 (all of the included studies), 5 [40, 42], 6 [37, 39, 40], 8 [45], 10 [10, 27, 29, 31, 32, 34, 36-46], 12 [10, 27, 30, 32, 34, 38, 41, 43, 44, 46], 14 [31, 32, 36, 39] and 16 [10, 29, 34, 36] (Table 3).

**Table 3:**
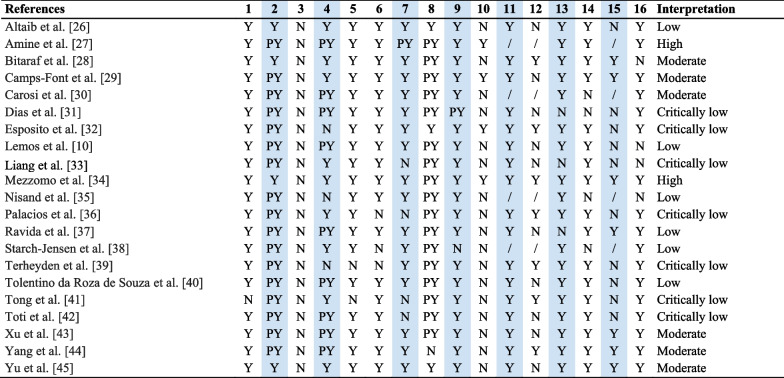
Confidence in methodological quality of included SR/MA according to AMSTAR-2 checklist

Critical items according to AMSTAR-2 checklist

**Fig. 2.**
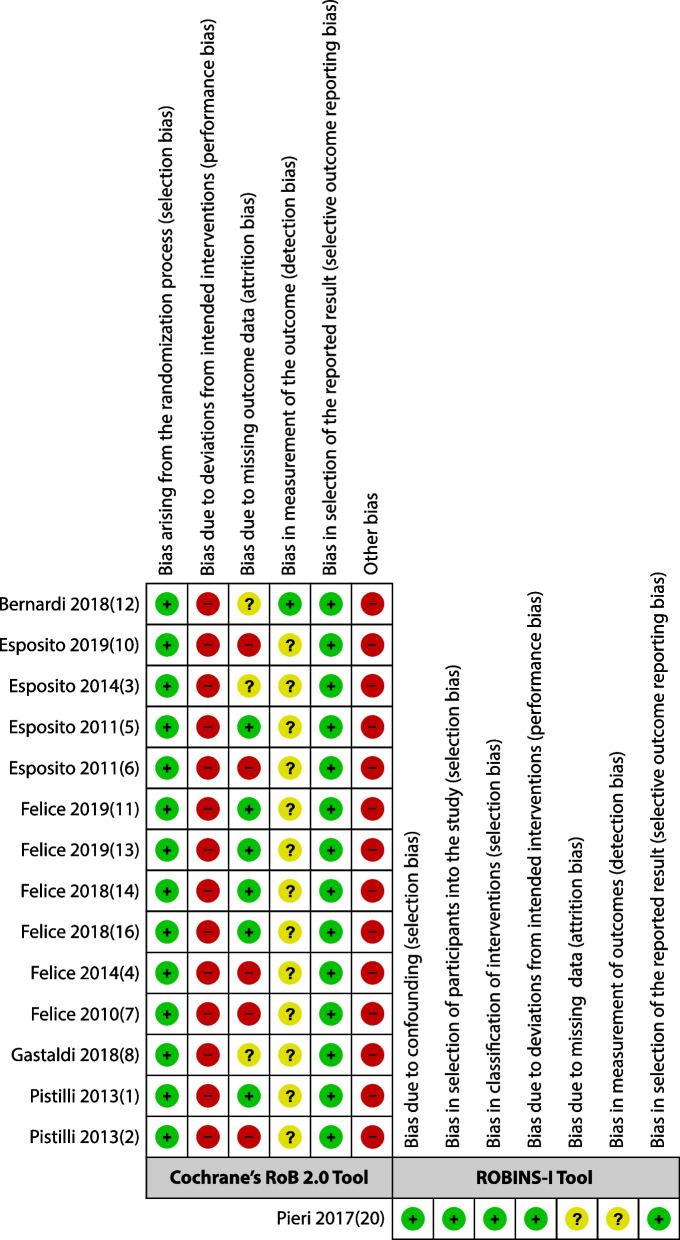
Results of the risk of bias assessment based on the Cochrane RoB 2.0 tool for RCT and ROBINS-I for nonrandomized studies of interventions (Green: low risk, yellow: medium risk, and red: high risk)

Regarding the RCTs assessed using the Cochrane RoB 2.0 Tool, the results of all studies were mostly with a “Low” risk of bias for the domain “Bias arising from the randomization process”, because they used a coin toss [47] or a computer-generated restricted randomized list [48-60]. The domain “Bias arising from deviations from intended interventions” was assessed as “High” in all studies, because participants and surgeons were aware of the intervention and control group during the trial [47], an inevitable limitation for ethical reasons. In addition, some cases presented deviations in the intended interventions; in the control group, as they failed or the BA materials were unavailable [53], it was decided to change the intervention, combine the interventions [48], or change between the groups [56, 60]. These deviations were partially unbalanced and could affect the results of all outcomes because the sample size was small. For “Bias due to missing outcome data”, studies assessed as “Low”, had no loss to follow-up [54]. However, in the studies where participants withdrew or could not be located (“loss to follow-up” or “dropout”), and participants who did not attend the visit at which outcomes should have been measured, the reasons were not related to the true nature of the intervention (change in residency) [48-50, 54, 56, 60]; in addition, they used complete case datasets. Although the studies evaluated as “High”, although used complete case data sets, there is evidence that the reasons for dropouts could be related to the true nature of the interventions, i.e., lack of motivation to attend maintenance sessions for the augmented bone group, due to their multiple failure procedures (adherence to follow-up) [51, 53, 57-59]. Of concern were the differences in the analysis and reporting of data from two studies included in the threads, evaluating the same cohort at different follow-ups [52, 55], and incomplete reporting of the prosthesis analysis at the 1-year follow-up [47]. In this case, it was decided to use the first data reported in the study thread (considering individual studies) for the MBL outcome; for the other outcomes, the data reported in the first published article of the study threads were used. The unavoidable lack of blinding of the evaluators did not imply a severe limitation for all results. Thus, we considered “Low” risk of bias for the evaluation of Implant and Prosthesis Failure and Biological complications, due to their binary nature [47]. In contrast, for the assessment of MBL, concerns were raised owing to the obvious radiological differences between native and augmented bone, which could influence the outcome measurement. For “Bias in selection of reported outcomes”, the results of all studies were assessed as “Low”, because there was no evidence of discordance between the pre-specified data analysis and the reported outcomes (multiple analysis selection), for all outcomes. Finally, for “Other biases”, all included studies were assessed as “High”, because they had neither a registered protocol for prospective RCTs nor adequate sample size calculation. In addition, some concerns about funding may arise, even if the authors stated no conflict of interest [47-60] (Fig. 2).

For the included retrospective cohort study [61], assessed with ROBINS-I, the results for “Confounding bias”, “Bias in study participant selection”, and “Bias in intervention ratings” were assessed as “Low”, as the pre-intervention prognostic factors, such as bone thickness, were almost entirely equivalent to those of the RCTs included in this review and the population was balanced in all other respects, such as its characteristics and risk factors; all participants who were eligible for the target trial were included in the study, and for each participant, the start of follow-up and the start of the intervention coincided. Their status was well-defined, based solely on information collected at the time of the intervention (medical records). Regarding “implementation bias”, there was no evidence of any deviation from the intended interventions (low risk of bias). Regarding “bias due to missing outcome data,” the results were assessed as “moderate” due to some concerns related to the unbalanced dropout rate of the intervention and control groups (4 vs. 2 participants, respectively). However, they performed a complete case data analysis. There was a “Moderate” risk of bias for all outcomes, as participants, surgeons, and evaluators were aware of the interventions. However, in the case of MBL, obvious differences could have influenced the outcomes. Finally, there was no evidence of selective outcome reporting, as prespecified outcomes were reported in full in the results (low risk of bias) (Fig. 2).

### Effects of interventions and summary of findings

**Fig. 3.**
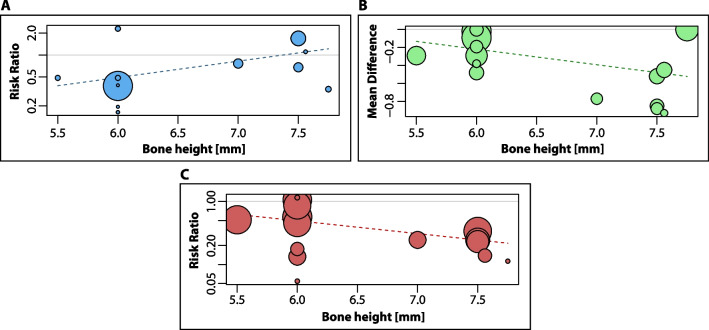
Exploratory meta-regression analysis. **A** The Bubble Plot does not show a relationship with the risk of implant failure, from 1 to 8 years of follow-up. **B** The Bubble Plot shows that “bone height” is associated with MBL, from 1 to 8 years of follow-up. **C** The Bubble Plot shows that “bone height” is associated with the risk of biological complications, from 1 to 8 years of follow-up

The main results from the evidence synthesis regarding the SR/MA included (Table 4), the meta-analysis of the primary studies (Additional file 1: Figs. S1–S4) and meta-regression analysis for the primary outcomes (Fig. 3) are presented in the following table.

**Table 4:** Summary of findings table

Outcomes	Follow-up/subgroups		Sample size* (studies)	Relative effect (95% CI)	Anticipated absolute effects**		Certainty of the evidence (GRADE)
					Risk with regular dental implants and bone augmentation	Risk difference with short dental implants in native bone	
Implant level							
Implant failure (before and after loading)	1 year		498 (5 RCTs)	RR 0.33 (0.15 to 0.74)	87 per 1000 implants	29 fewer per 1000 implants	⨁⨁◯◯ Low^a,e^
					Difference: 58 fewer per 1000 implants (74 to 23 fewer)		
	3 years		328 (4 RCTs)	RR 0.68 (0.22 to 2.06)	53 per 1000 implants	36 per 1000 implants	⨁◯◯◯ Very low^a,d,e^
					Difference: 17 fewer per 1000 implants (42 fewer to 56 more)		
	5 years		425 (4 RCTs, 1 NRSI)	RR 1.18 (0.50 to 2.79)	45 per 1009 implants	53 per 1000 implants	⨁◯◯◯ Very low^a,d,e^
					Difference: 8 more per 1000 patients (23 fewer to 81 more)		
	8 years		121 (1 RCT)	RR 1.69 (0.42 to 6.78)	49 per 1000 implants	83 more per 1000 implants	⨁◯◯◯ Very low^a,d,e^
					Difference: 34 more per 1000 patients (29 fewer to 284 more)		
Marginal bone loss (change from baseline)	1 year		209 (4 RCTs, 1 NRSI)	–	The mean MBL ranged from 0.75 to 1.25 mm	MD 0.07 mm lower (0.14 lower to 0.01 higher)	⨁◯◯◯ Very low^a,b,c,e,g^
	3 years		142 (4 RCTs)	–	The mean MBL ranged from 1.39 to 1.76 mm	MD 0.32 mm lower (0.44 lower to 0.19 lower)	⨁⨁◯◯ Low^a,f^
	5 years	Short dental implant length of 4 to < 6 mm	44 (2 RCTs)	-	The mean MBL ranged from 1.7 to 2.1 mm	MD 0.45 mm lower (0.72 lower to 0.18 lower)	⨁⨁◯◯ Low^a,f^
		Short dental implant length of 6 to 8.5 mm	119 (2 RCTs, 1 NRSI)	–	The mean MBL ranged from 1.61 to 2.34 mm	MD 0.84 mm lower (1.07 lower to 0.61 lower)	⨁⨁◯◯ Low^a,f^
	8 years	Short dental implant length of 6 to 8.5 mm	47 (1 RCT)	–	The mean MBL was 2.46 mm	MD 0.88 mm lower (1.26 lower to 0.5 lower)	⨁⨁◯◯ Low^a,f^
Biological complications (before and after loading)	1 year	Short dental implant length of 4 to < 6 mm	119 (2 RCTs)	RR 0.48 (0.25 to 0.93)	295 per 1000 implants	142 per 1000 implants	⨁◯◯◯ Very low^a,d,e^
					Difference: 153 fewer per 1000 implants (221 fewer to 21 fewer)		
		Short dental implant length of 6 to 8.5 mm	379 (3 RCTs)	RR 0.12 (0.04 to 0.32)	188 per 1000 implants	23 per 1000 implants	⨁⨁⨁◯ Moderate^a^
					Difference: 165 fewer per 1000 implants (180 fewer to 128 fewer)		
	3 years	Short dental implant length of 4 to < 6 mm	119 (2 RCTs)	RR 0.66 (0.39 to 1.09)	475 per 1000 implants	314 per 1000 implants	⨁⨁◯◯ Very low^a,d,e^
					Difference: 161 fewer per 1000 patients (290 fewer to 43 more)		
		Short dental implant length of 6 to 8.5 mm	209 (2 RCTs)	RR 0.21 (0.10 to 0.46)	324 per 1000 implants	68 per 1000 implants	⨁⨁⨁◯ Moderate^a^
					Difference: 256 fewer per 1000 implants (292 fewer to 175 fewer)		
	5 years	Short dental implant length of 4 to < 6 mm	119 (2 RCTs)	RR 0.77 (0.42 to 1.42)	475 per 1000 implants	366 per 1000 implants	⨁◯◯◯ Very low^a,d,e^
					Difference: 109 fewer per 1000 implants (276 fewer to 200 more)		
		Short dental implant length of 6 to 8.5 mm	306 (2 RCTs, 1 NRSI)	RR 0.22 (0.12 to 0.40)	346 per 1000 implants	76 per 1000 implants	⨁⨁⨁◯ Moderate^a^
					Difference: 270 fewer per 1000 implants (304 fewer to 208 fewer)		
	8 years	Short dental implant length of 6 to 8.5 mm	121 (1 RCT)	RR 0.34 (0.17 to 0.66)	443 per 1000 implants	151 per 1000 implants	⨁⨁⨁◯ Moderate^a^
					Difference: 292 fewer per 1000 implants (367 fewer to 150 fewer)		
Prosthetic level							
Prosthesis failures and complications	3 years		154 (4 RCTs)	RR 0.65 (0.23 to 1.84)	117 per 1000 implants	76 per 1000 implants	⨁◯◯◯ Very low^a,d,e^
					Difference: 41 fewer per 1000 patients (90 fewer to 98 more)		
	5 years		176 (4 RCTs, 1 NRSI)	RR 0.91 (0.45 to 1.84)	161 per 1000 implants	147 per 1000 implants	⨁◯◯◯ Very low^a,d,e^
					Difference: 14 fewer per 1000 patients (89 fewer to 135 more)		
	8 years		48 (1 RCT)	RR 1.23 (0.31 to 4.90)	130 per 1000 implants	160 per 1000 implants	⨁◯◯◯ Very low^a,d,e^
					Difference: 30 more per 1000 patients (90 fewer to 509 more)		
Patient satisfaction							
Patient’s treatment preference side (split-mouth)	1-month post-loading		40 (1 RCT)	One month after delivery of the definitive prostheses, an independent assessor asked the patients which treatment they preferred. All 20 patients treated with mandibular implants preferred short implants side vs conventional length implants placed in augmented bone side (*p* < 0.0001)			⨁⨁◯◯ Low^a,e^
Cost-effectiveness	There is no data regarding the costs, or other economic evaluation that assess the focused question of the present umbrella review						

Patient or population: atrophic mandible

Setting: private dental clinic—Italy

Intervention: short dental implants in native bone

Comparison: regular dental implants and bone augmentation

GRADE Working Group grades of evidence—high certainty: we are very confident that the true effect lies close to that of the estimate of the effect. Moderate certainty: we are moderately confident in the effect estimate: the true effect is likely to be close to the estimate of the effect, but there is a possibility that it is substantially different. Low certainty: our confidence in the effect estimate is limited: the true effect may be substantially different from the estimate of the effect. Very low certainty: we have very little confidence in the effect estimate: the true effect is likely to be substantially different from the estimate of effect

*CI* confidence interval, *MD* mean difference, *NRSI* non-randomized study of intervention, *RCT* randomized controlled trial, *RR* risk ratio

Explanations

Risk of bias: ^a^High risk of bias studies largely contribute to the weighted overall estimate of effect due to the lack of blinding of participants, surgeons and evaluators. We decided to downgrade only one level, as we recognized the impossibility to blind the intervention and comparison (evident differences between implant lengths). In addition, we did not downgrade due to the lack of sample size calculations (high risk of other bias), as we could perform a post-hoc power analysis for the meta-analysis using TSA

Inconsistency: ^b^Sensitivity analysis: the source of heterogeneity may be due to the differences of the risk of bias between Pieri 2017 (moderate risk of bias) and the other RCTs (high risk of bias). We decided to downgrade one level because of this. ^c^There is a statistically significant moderate to high heterogeneity (assessed with *I*^2^ and *p* value). Therefore, we decided to downgrade one level

Indirectness: we do not have serious concerns regarding the indirect evidence or clinical diversity of the included studies (PICO)

Imprecision: ^d^Wide confidence intervals that include “no effect” and appreciable benefits and harms (RR less than 0.75 or over 1.25). Therefore, we decided to downgrade two levels. ^e^The available number of events does not allow to reach the calculated Optimal Information Size. Therefore, we decided to downgrade one level. ^f^Although the OIS is reached by power calculations, and in consequence, is sufficient to detect statistically significant differences, we decided to downgrade one level because there is not a minimal important difference reported in the literature that allows an adequate clinical difference estimate. ^g^Wide confidence intervals that include “no effect” and appreciable benefits and harms (minimal clinical difference set to “MD different from zero”). Therefore, we decided to downgrade one level

Publication bias: we do not have serious concerns regarding the publication bias (funnel plot observation)

*Participants or implants

**The risk in the intervention group (and its 95% confidence interval) is based on the assumed risk in the comparison group and the relative effect of the intervention (and its 95% CI)

### Implant failure

Of the SR/MA included, three concluded that at 1, 5 and 10 years, there was a lower risk of implant failure in the short implant group [31, 32, 45]. On the other hand, three studies reported that at 1, 5, 8, and 10 years, regular implants with bone augmentation had a lower risk of implant failure [35, 44, 46]. However, a total of 15 MAs showed that at 4 months, 1, 2, 3, 5, 8 and 10 years, there was no significant difference between the risk of implant failure between the short implant group and the regular implant group with bone augmentation [10, 27-30, 33, 34, 36-43]. Furthermore, the results of the raw meta-analytic model of 15 studies [47-61] (Additional file 1: Fig. S1) showed no differences between the groups (RR 0.70, 95% CI 0.43–1.13, *p* = 0.14; OIS = 1372 of 4519 implants), with no evidence of heterogeneity between studies (*I*^2^ = 0%, *p* = 0.75). However, the results of the subgroup analysis showed an interaction between follow-up and implant failure (*I*^2^ = 52.2%, *p* = 0.10), but not for short implant length (*I*^2^ = 0%, *p* = 0.81). Nevertheless, the results for the 4 to < 6 mm group (53–58) (RR 0.78, 95% CI 0.28–2.18, *p* = 0.63, OIS = 357 of 11,249; *I*^2^ = 0%, *p* = 0.70) and for the 6 to 8 mm group (RR 0.67, 95% CI 0.39–1.17, *p* = 0.16, OIS = 1015 of 3379 implants; *I*^2^ = 0%, *p* = 0.53) (49–52, 60, 61, 63) showed no differences between the groups. Thus, the results presented by follow-up showed that:

- At 1 year of follow-up [47, 48, 51, 54, 57], there was a lower risk of implant failure in favor of short implants (RR 0.33, 95% CI 0.15–0.74, *p* = 0.007, OIS = 498 out of 505 implants; *I*^2^ = 0%, *p* = 0.98) (Additional file 1: Fig. S1a).
- At 3 years of follow-up [49, 52, 55, 58], there was no difference between the groups (RR 0.68, 95% CI 0.22–2.06, *p* = 0.49, OIS = 328 out of 4639 implants; *I*^2^ = 0%, *p* = 0.56) (Additional File 1: Fig. S1b).
- At 5 years of follow-up [50, 53, 56, 59, 61], there was no difference between the groups (RR 1.18, 95% CI 0.50–2.79, *p* = 0.71, OIS = 425 out of 11,912 implants; *I*^2^ = 0%, *p* = 0.85) (Additional file 1: Fig. S1c).
- At 8 years of follow-up [60], there was no difference between the groups (RR 1.69, 95% CI 0.42–6.78, *p* = 0.46, OIS = 121 out of 1686 implants) (Additional file 1: Fig. S1d).

### Marginal bone loss

Of the SR/MA included, 14 concluded that at 1, 2, 3, 5, 8, and 10 years, the short implant group had less MBL compared to the regular implant group [28, 29, 31, 33, 36, 38-46]. Only one MA reported less MBL in the regular implant group with BA at 1 year [35]. On the other hand, five MA concluded that at 4 months and 1, 2, 3, 5, 8 and 10 years, there was no significant difference between these 2 groups [10, 27, 30, 34, 37]. The raw meta-analysis of 14 studies [48-61] showed a reduction in MBL of 0.33 mm, in favor of short dental implants (95% CI − 0.43 to − 0.22 mm, *p* < 0.00001, OIS = 561 of 138 patients) with high between-study heterogeneity (*I*^2^ = 86%, *p* < 0.00001). In this regard, subgroup analysis showed an interaction between follow-ups (*I*^2^ = 94.2%, *p* < 0.00001) with MBL, but not for short implant length (*I*^2^ = 32.2%, *p* = 0.22) (Additional file 1: Fig. S2). However, the overall effect for the 4 to < 6 mm group [51-56] is a reduction of 0.20 mm (95% CI − 0.34 to − 0.05, *p* = 0.007, OIS = 170 of 233 patients; *I*^2^ = 49%, *p* = 0. 08) and a reduction of 0.48 mm for the 6 to 8.5 mm group [48-50, 58, 59, 61], in favor of short implants (95% CI − 0.71 to − 0.24, *p* < 0.0001, OIS = 391 of 212 patients; *I*^2^ = 91%, *p* < 0.00001). Hence, the results by follow-up showed that:

- At 1 year of follow-up, there was no difference between the groups (MD − 0.07 mm, 95% CI − 0.14 to 0.01 mm, *p* = 0.08; *I*^2^ = 70%, *p* = 0.01, OIS = 209 out of 490 patients) [48, 51, 54, 57, 61] (Additional file 1: Fig. S2a).
- At 3 years of follow-up, the MBL was 0.32 mm (95% CI − 0.44 to − 0.19 mm, *p* < 0.00001, OIS = 142 out of 46 patients; *I*^2^ = 0%, *p* = 0.50) that favors short dental implants [49, 52, 55, 58] (Additional file 1: Fig. S2b).
- At 5 years of follow-up, the MBL was 0.45 mm (95% CI − 0.72 to − 0.18 mm, *p* = 0.001, OIS = 44 out of 33 patients; *I*^2^ = 0%, *p* = 0.75) that favors the group of short dental implant length of 4 to < 6 mm [53, 56] (Additional file 1: Fig. S2c.1), and 0.84 mm (95% CI − 1.07 to − 0.61, *p* < 0.00001, OIS = 119 out of 19 patients; *I*^2^ = 0%, *p* = 0.89) that favors the group of short dental implant length of 6 to 8.5 mm [50, 59, 61] (Additional file 1: Fig. S2c.2) (subgroup differences, *I*^2^ = 77.7%, *p* = 0.03) (Additional file 1: Fig. S2c).
- At 8 years of follow-up, the MBL was 0.88 mm (95% CI − 1.26 to − 0.5, *p* < 0.00001, OIS = 47 out of 18 patients) that favors the group of short dental implant length of 6 to 8.5 mm [60] (Additional file 1: Fig. S2d).

### Biological complications (before and after loading)

Of the SR/MA included, nine reported that at 1, 2, 3, 5, 8, and 10 years there was a lower risk of biological complications in the short implant group [29-31, 36, 38, 41-44]. However, only two studies concluded that at 4 months and 1 year, there were no significant differences between the two groups [10, 35]. The raw meta-analysis of 15 studies [47-61], that reported biological complications as mucositis and peri-implantitis, showed a relative risk reduction (RRR) of 63% (RR 0.37, 95% CI 0.26–0.54, *p* < 0.00001, OIS = 1372 out of 361 implants) in favor of short dental implants, with evidence of moderate heterogeneity (*I*^2^ = 57%, *p* = 0.004). Therefore, the results of the subgroup analysis showed no interaction between follow-up and biological complications (*I*^2^ = 0%, *p* = 0.84), in contrast to the evident interaction with short dental implant length (*I*^2^ = 95.1%, *p* < 0.00001). This leads to an RRR of 34% that favors the 4 to < 6 mm group (RR 0.66, 95% CI 0.50–0.88, *p* = 0.005, OIS = 357 out of 356 implants; *I*^2^ = 0%, *p* = 0.43) [51-56] and 77% that favors the 6 to 8.5 mm group (RR 0.23, 95% CI 0.16–0.33, *p* < 0.00001, OIS = 1015 out of 91 implants; *I*^2^ = 0%, *p* = 0.83) [47-50, 58, 59, 61]. Despite the lack of interaction between follow-ups, the results of this covariate are summarized as follows:

- At 1 year of follow-up, there was a lower risk of biological complications that favors short dental implants (RR 0.48, 95% CI 0.25–0.93, *p* = 0.03, OIS = 119 out of 228 implants; *I*^2^ = 0%, *p* = 0.51) [51, 54] (Additional file 1: Fig. S3a).
- At 3 years of follow-up, there was no difference between groups (RR 0.66, 95% CI 0.39–1.09, *p* = 0.11, OIS = 119 out of 344; *I*^2^ = 0%, *p* = 0.27) [52, 55] (Additional file 1: Fig. S3b).
- At 5 years of follow-up, there was no difference between groups (RR 0.77, 95% CI 0.42–1.42, *p* = 0.40, OIS = 119 out of 1048; *I*^2^ = 48%, *p* = 0.16) [53, 56] (Additional file 1: Fig. S3c).

For the subgroup “short dental implant length of 6 to 8.5 mm:

- At 1 year of follow-up, there was a lower risk of biological complications that favors short dental implants (RR 0.12, 95% CI 0.04–0.32, *p* < 0.0001, OIS = 379 out of 109 implants; *I*^2^ = 0%, *p* = 0.84) [47, 48, 57] (Additional file 1: Fig. S3a).
- At 3 years of follow-up, there was a lower risk of biological complications that favors short dental implants (RR 0.21, 95% CI 0.10–0.46, *p* < 0.0001, OIS = 209 out of 76 implants; *I*^2^ = 0%, *p* = 0.75) [49, 58] (Additional file 1: Fig. S3b).
- At 5 years of follow-up, there was a lower risk of biological complications that favors short dental implants (RR 0.22, 95% CI 0.12–0.40, *p* < 0.001, OIS = 306 out of 72 implants; *I*^2^ = 0%, *p* = 0.77) [50, 59, 61] (Additional file 1: Fig. S3c).
- At 8 years of follow-up, there was a lower risk of biological complications that favors short dental implants (RR 0.34, 95% CI 0.17–0.66, *p* = 0.001, OIS = 121 out of 77 implants) [60] (Additional file 1: Fig. S3d).

### Prosthesis failures and complications

Of the SR/MA included, six concluded that at 4 months and 1, 2, 3, and 5 years, there was no significant difference in the risk of technical complications between the short implant group and the regular implant group with BA [30, 36-38, 42, 43]. On the other hand, three studies reported that at 1, 5, 8, and 10 years, there was a lower risk for regular implants with BA [31, 35, 44]. In contrast, one study concluded that there was a lower risk in the short implant group at 1, 3, and 5 years [41]. The raw meta-analysis of ten studies [49, 50, 52, 53, 55, 56, 58-61], which included prosthetic complications such as ceramic chipping, decementation and abutment loss, showed an RRR of 13% for prosthetic failures and complications for short implants (RR 0.87, 95% CI 0.51–1.49, *p* = 0.62, OIS = 378 out of 2787 patients), with evidence of no heterogeneity across the studies (*I*^2^ = 0%, *p* = 0.87) (Additional file 1: Fig. S4). The subgroup analysis revealed an interaction between follow-up, short implant length and risk of prosthesis failures and complications (*I*^2^ = 0%, *p* = 0.76; and *I*^2^ = 0%, *p* = 0.85, respectively). In summary, the results by follow-up are presented as follows:

- There was no difference between groups at 3 (RR 0.65, 95% CI 0.23–1.84, *p* = 0.42, OIS = 154 out of 1635 patients; *I*^2^ = 0%, *p* = 0.54) [49, 52, 55, 58] (Additional file 1: Fig. S4), 5 (RR 0.91, 95% CI 0.45–1.84, *p* = 0.80, OIS = 176 out of 2351 patients; *I*^2^ = 0%, *p* = 0.76) [50, 53, 56, 59, 61] (Additional file 1: Fig. S4b) and 8 (RR 1.23, 95% CI 0.31 to 4.90, *p* = 0.77, OIS = 48 out of 3698) [60] years of follow-up (Additional file 1: Fig. S4c).

Among the SR/MA that mixed biological and technical/prosthetic complications, it was reported that at 1, 3, 5 and 10 years the risk of complications was lower in the short implant group [27, 33, 39, 40, 45, 46]. However, one reported that at 1 year there was less risk in the group of regular implants with BA [32].

### Patient-reported outcome measures and other outcomes

The results of an RCT of 40 hemiarches (split-mouth) [48] reported that 1 month after delivery of the final prostheses, an independent evaluator asked patients which treatment they preferred. All 20 patients treated with mandibular implants preferred the short implant side versus conventional length implants placed on the augmented bone side (*p* < 0.0001) (low certainty of evidence) (Table 4). In addition, there were no data on other outcomes or cost-effectiveness analyses comparing the intervention and control groups.

### Meta-regression analysis

Meta-regression exploratory analysis assessing the effect of the predictor “bone height” did not show a relationship with the risk of implant failure (estimate = 0.52, 95% CI − 0.12 to 1.16, *p* = 0.109, R2 = 0%) (Fig. 3A). However, it showed that “bone height” predicted MBL (estimate = − 0.17, 95% CI − 0.3 to − 0.05, *p* = 0.007, R2 = 21.15%) (Fig. 3B) and the risk of biological complications (estimate = − 0.47, 95% CI − 0.87 to − 0.07, *p* = 0.022, R2 = 49.64%) (Fig. 3C) from 1 to 8 years of follow up.

### Publication bias

Observation of the funnel plots for the raw meta-analytic models showed no asymmetry indicating publication bias for the studies included in the outcomes “implant failure” (Additional file 1: Fig. S6A) and “prosthetic failure and complications” (Additional file 1: Fig. S6D), which was confirmed by the Peters test for funnel plot asymmetry (*p* = 0.565 and *p* = 0.295, respectively). In contrast, for the outcomes “MBL” and “biological complications,” the observation of the funnel plot showed asymmetry (Additional file 1: Fig. S6C, D), as confirmed by Egger’s (*p* = 0.0004) and Peters’ (*p* = 0.0013) tests, respectively. However, when the available studies were analyzed by subgroup, there were not enough studies to make an optimal funnel plot observation (≤ 5), although no biased results were suspected for those subgroups.

## Discussion

In summary, the results of the meta-analysis showed that short implants were placed in the native bone, compared to regular length implants, placed after BA in the posterior atrophic mandible.

- Implant failure (before and after loading): less implant failure at 1 year follow-up. Nevertheless, the evidence is very uncertain regarding the true effect at 3, 5, and 8 years of follow-up.
- Marginal bone loss (change from baseline): the evidence is very uncertain regarding the true effect at 1 year of follow-up. However, this may result in a reduction of the MBL at 3, 5, and 8 years of follow-up.
- Biological complications (before and after loading): the evidence is very uncertain regarding the true magnitude of the effect at 1, 3, and 5 years of follow-up for the 4 to < 6 mm group. At the same time, for the 6–8.5 mm group, the risk of biological complications is reduced at 1, 3, 5 and 8 years of follow-up.
- Prosthetic failure and complications: the evidence is very uncertain regarding the magnitude and direction of the true effect at 3, 5 and 8 years of follow-up.
- Patient preference: the patients may prefer short dental implants when compared to the control group.
- Cost-effectiveness: there are no data regarding the costs, or other economic evaluations that assess the focused question of the present UR.

In addition, it partially clarifies when one intervention might be more predictable than another. In this sense, the meta-regression analysis is consistent with the literature, indicating that up to ~ 4 mm, there are no significant differences between BA techniques [4]. As the basal bone height increases, and consequently the height to be augmented decreases, the risk of biological complications and MBL associated with the use of regular implants placed in augmented bone also decreases. In this sense, the associated risks are more related to BA surgery than to implant length. On the other hand, three-dimensional finite analysis models have shown that implant diameter, bone type and location (premolar) would be the most important factors in predicting bone stress, because the greatest stress occurs in the mandibular alveolar ridge and not in the apical peri-implant portion, concluding that the larger the diameter and the better the bone quality (thicker cortex), the lower the stress [62, 63]. At the same time, the larger the diameter, the larger the implant–bone contact area, and consequently, the surface area for osseointegration [64].

These results are in line with those described by Vetromilla et al., who compared short implants to regular implants plus sinus floor elevation and found a better or equal performance in implant survival (high certainty), MBL, biological complications, prosthetic outcomes (moderate certainty), and patient satisfaction [65], with a much lower cost, favoring short implants. However, the evidence from which the primary data were collected is “critically low” or “low”, so that SR/MA are required to allow a better synthesis of the evidence [66].

Regarding the assessment of the body of evidence, for the risk of bias of the included studies, for all outcomes, studies with high risk of bias contributed greatly to the overall weighted estimate of effect, due to the lack of blinding of participants, surgeons, and evaluators. It was decided to downgrade only one level, as the impossibility of blinding the intervention and comparison (obvious differences between implant lengths) was recognized. In addition, the level was not downgraded due to the lack of sample size calculations, as a post-hoc power analysis could be performed for the meta-analysis using TSA. No serious concerns were raised regarding the inconsistency of the results, as they did not demonstrate significant evidence of heterogeneity among the included studies, except for the 1-year MBL. In this case, a sensitivity analysis was performed for risk of bias and study design showing a minimal reduction in accounted heterogeneity from *I*^2^ = 70% (*p* = 0.010) to *I*^2^ = 55% (*p* = 0.08) and overall effect (absolute ∆MD = 0.02 mm). For this reason, it was decided to downgrade one level.

There were no serious concerns regarding indirect evidence or clinical diversity in the studies included in our meta-analysis (PICO), even if there is one low-weight study (< 25%) that featured short implants placed in augmented bone, due to the lack of installation of a regular-length implant [56]. In addition, most of the included SRs/MAs reported mixed maxillary/mandibular or native/augmented bone data, relative to the results of their primary and secondary outcomes. This resulted in a problem of indeterminacy of evidence in the aforementioned synthesis results, i.e., for the biomechanical properties of the bone itself [67]. Regarding imprecision, it was decided to downgrade 2 levels in the studies with wide confidence intervals which included “no effect” and appreciable benefits and harms (RR less than 0.75 or greater than 1.25, or minimum clinical difference established as “MD different from zero”). In addition, it was decided to downgrade one level when the number of events available did not allow the calculated OIS to be reached, or when, although the OIS is reached by power calculations, there was no important minimum difference reported in the literature that would allow an adequate estimate (MBL): for short implants the MBL is more detrimental compared to the same MBL for regular implants.

Finally, there were no serious doubts about publication bias, as the limited number of studies included in the meta-analysis by follow-up and/or the short duration of dental implants did not allow an ideal visual or statistical assessment of the funnel plot to determine a possible publication bias among the included studies.

The present review is the first of its kind in the field and is not free of limitations, which also stem from the studies included in it. In relation to the methodological design of the present review, the use of the few available recommendations for the development of UR reduces reporting bias and thus provides reproducibility and transparency to the report. Second, the use of multiple search languages, databases, registries, and relevant gray literature allows for the collection of varied information, partially controlling for publication bias. The fact that most of the included SR/MA were considered as with “critically low” or “low” confidence allowed data to be extracted from the primary studies and not from the syntheses themselves, which brings accuracy to the development of meta-analytic models. The systematic and eminently clinical, patient-centered approach provided by GRADE also facilitates the interpretation of quantitative and qualitative evidence within the framework of what is available and guides the clinician to make fully informed decisions compatible with his or her own experience.

Regarding the methodological design of the included studies, it is important to emphasize that none of the included primary studies performed a sample size calculation. Although the split-mouth design has the advantage of removing a lot of inter-subject variability from the estimated treatment effect, it is more reliable when performed in a properly calculated population [68]. In addition, the risk of bias in most of the included studies was high, not unlike that reported in the discipline [69], not only because of the absence of sample size calculation but also because of problems in the blindness of the evaluator and the participant. Although RCTs are the gold standard for determining the effectiveness of interventions, one way to overcome the inherent limitations mentioned above, reduce bias, and provide greater external validity to the conclusions, could be the development of real-world parallel-arm cohort studies. At the same time, within the raw meta-analytic models, there was publication bias for MBL and biological complications, which could be influenced by the “funding” provided by the brands facilitating the implants and material required to perform the primary studies included [70]. However, this possible bias is given by the greater publication of articles of European origin [71-73]. More studies, mainly from other countries, are necessary for a more comprehensive assessment of publication bias and its effect on the reporting of results.

Regarding the population included in the primary studies, several important considerations should be taken into account for future studies, such as demographic factors [74] or the baseline oral health status of the patient. First, more thorough evaluations of the demonstrated risk factors for success, implant failure, MBL, and prosthetic failure, such as smoking [75-78], bruxism [79], history of periodontitis [80, 81], and peri-implant supportive therapy [82], which add bias in the presented results, should be performed. In view of the above, although smoking was balanced between the two groups, the outcomes of success could be explained by these covariates. Likewise, some studies pointed out that factors such as bruxism or periodontitis are relevant when analyzing implant survival, MBL, and associated complications, so their transparent reporting is important. When analyzing the results of the included primary studies, these last two factors are poorly reported and were only pointed out in the observational study [61]. Similarly, regarding the interventions, surgical experience [80] was ruled out as a significant factor in the outcomes evaluated, given that in the primary studies included, the procedures were performed by experienced surgeons. However, there was poor reporting of the quality and type of bone in which the surgeries were performed. This could have an impact on the results since the lower the bone quality, the lower the osseointegration [83, 84]. Despite the varied opinions on the subject [85], the use of a minimum effective dose of antibiotic prophylaxis [86] to prevent early local infections after implant installation [87], and consequently, implant failure [88, 89], is interesting. However, it was not specifically recorded whether the implants that failed were placed on sites where another implant had previously failed [90] or on an infected site [91]. Studies that also compare one- and two-stage bone augmentation surgeries [80] are needed to assess whether loading time interacts with success outcomes [92-94] in the context of these interventions to accurately measure the impact on the effect.

Regarding the management of peri-implant soft tissues, the condition of the peri-implant mucosa was not evaluated or reported, which is important because short implants are known to have a higher prevalence of mucositis in the mandible [95]. Other aspects not reported or evaluated in this review, such as the emergence profile and angle, may influence the occurrence of peri-implantitis [96], which, together with its sequelae, could compromise functionality, esthetics, and patient satisfaction.

Concerning prosthetic aspects, a report and evaluation of splinting, as well as its characteristics, is required, as it further decreases bone stress, and consequently can improve predictability, impacting on the magnitude of the overall effect [9, 95, 97-99]. Similarly, the use of internal connections [100] and smaller diameters (platform switching) [101] could have a beneficial effect on MBL. The type of prosthesis retention also needs to be evaluated, as cement-retained prostheses show less failure and MBL than screw-retained prostheses [102, 103], a factor that was not considered in the present study. Finally, the crown-to-implant ratio should be considered to accurately determine its impact on MBL, which also contributes to clarify the available evidence [104, 105]: at the moment, only one study reported this parameter.

In relation to the satisfaction and experience of the patients who were included in the reviewed studies, this was not evaluated or reported under any validated scale or any other report, but it has been observed that patients who receive short implant treatments have greater satisfaction according to the OHIP-14 scale [106]. On the other hand, patient satisfaction was not considered when selecting the type of graft to be used, which is relevant in this type of procedure, since xenografts present less postoperative pain and operating time, which could mean an increase in patient satisfaction [107, 108]. Complications associated with BA surgery occur frequently [3, 4], including paresthesia of the inferior alveolar nerve [57], which leads to evident discomfort in patients, and is considered a complication of low relevance, but which could be important in the healing period and should be taken into account when making clinical decisions.

Consequently, RCTs and real-world evidence are needed to use the aspects mentioned as adjustment covariates for more complex statistical models, such as multi-level, hierarchical, frequentist, Bayesian meta-analysis, or for individual-patient data meta-analysis, generating results that could be evaluated economically. In addition, it is also necessary to gather more information from the patient perspective. Both aspect are considered very underreported in the present review and in the literature of the discipline [109]. In addition, more research is needed on the impact of the interventions evaluated in the present review on peri-implantitis critical outcomes in terms of prevalence and incidence. However, there is an ongoing parallel RCT registry (NCT03524885) comparing short implants (4–5 mm) with regular implants (10–13 mm) plus GBR, which will add information on peri-implant bone-level changes, patient satisfaction, and implant survival at 1-year post-loading.

## Conclusions

The available evidence partially suggests that the use of short implants could decrease implant failure, MBL, and biological complications, while increasing patient satisfaction. However, given the need for further RCTs and real-world evidence to fully evaluate short- and long-term outcomes, it would be prudent for clinicians to carefully consider the patient needs and circumstances before deciding whether to use short implants.

## Supplementary Information

**Additional file 1.** Supplementary tables and figures.

## Data Availability

The data that support the findings of this study are available from the corresponding author, LD, upon reasonable request.

## Abbreviations

BA: Bone augmentation
CI: Confidence interval
MBL: Marginal bone loss
OIS: Optimal information size
SR/MA: Systematic reviews and meta-analysis
RCT: Randomized controlled trials
RR: Risk ratio
RRR: Relative risk reduction
TSA: Trial sequential analysis
UR: Umbrella review
