# Supplementary material for: Short implants compared to regular dental implants after bone augmentation in the atrophic posterior mandible: umbrella review and meta-analysis of success outcomes
Source: Int J Implant Dent. 2023 Jul 4;9:18. doi: 10.1186/s40729-023-00476-0 (PMC10317914; doi:10.1186/s40729-023-00476-0)
Supplement: Supplementary file 1 — Additional file 1. Supplementary tables and figures. [file 40729_2023_476_MOESM1_ESM.pdf]

Additional File 1

Table S1. Search queries

Databases and registries	Search algorithm	Filters	Results
**Cochrane Library**	(posterior mandible OR mandible OR posterior jaw OR inferior jaw OR partially edentulous jaw OR edentulous jaw OR Tooth Extraction OR Alveolar Bone Loss OR Tooth Loss) AND (shorter implant OR short dental implant OR extra short dental implant OR ultra short dental implant) AND ((Longer implant OR Normal implant OR standard implant OR Conventional implant OR Conventional length implant OR Dental Implant) AND (Bone Substitutes OR bone augmentation OR bone regeneration OR augmented bone OR ridge augmentation OR bone grafting))	With Cochrane Library publication date from Jan 2012 to May 2022, in Cochrane Reviews, Cochrane Protocols, Trials, Clinical Answers, Editorials and Special Collections (Word variations have been searched)	99 Trials (CENTRAL) 2 Cochrane reviews
**ClinicalTrials. gov**	(Longer implant OR Normal implant OR standard implant OR Conventional implant OR Conventional length implant OR Dental Implant) AND (Bone Substitutes OR bone augmentation OR bone regeneration OR augmented bone OR ridge augmentation OR bone grafting) | (posterior mandible OR mandible OR posterior jaw OR inferior jaw OR partially edentulous jaw OR edentulous jaw OR Tooth Extraction OR Alveolar Bone Loss OR Tooth Loss) | (shorter implant OR short dental implant OR extra short dental implant OR ultra short dental implant)	-	5
**EBSCOhost (Dentistry & Oral sciences source)**	("posterior mandible*" OR "mandible*" OR "posterior jaw*" OR "inferior jaw*" OR "partially edentulous jaw*" OR "edentulous jaw*" OR "Tooth Extraction" OR "Alveolar Bone Loss" OR "Tooth Loss") AND ("short* implant*" OR "short dental implant*" OR "extra?short dental implant*" OR "ultra?short dental implant*") AND (("Long* implant*" OR "Normal implant*" OR "standard implant*" OR "Conventional implant*" OR "Conventional length implant*" OR "Dental Implant*")) AND ("Bone Substitut*" OR "bone augment*" OR "bone regenerat*" OR "augmented bone" OR "ridge augmentation" OR "bone graft*"))	Year: 2012-2022	77
**LiLACS**	((posterior mandible) OR (mandible) OR (posterior jaw) OR (inferior jaw) OR (partially edentulous jaw) OR (edentulous jaw) OR (Tooth Extraction) OR (Alveolar Bone Loss) OR (Tooth Loss) OR (mandíbula posterior) OR mandíbula OR (desdentamiento parcial) OR (desdentamiento total) OR desdentado OR desdentada)) AND ((shorter implant) OR (short dental implant) OR (extra short dental implant) OR (ultra short dental implant) OR (implante corto) OR (implante ultra corto) OR (implante dental extra corto)) AND (((Longer implant) OR (Normal implant) OR (standard implant) OR (Conventional implant) OR (Conventional length implant) OR (Dental Implant) OR (implante dental) OR (implante convencional) OR (implante normal) OR (implante de largo estándar)) AND ((Bone Substitutes) OR (sustituto óseo) OR (bone augmentation) OR (bone regeneration) OR (augmented bone) OR (ridge augmentation) OR (bone grafting) OR (aumento óseo) OR regeneración ósea)))	-	0
**PubMED**	("posterior mandible*" OR "mandible*" OR "posterior jaw*" OR "inferior jaw*" OR "Jaw, Edentulous, Partially"[Mesh] OR "Jaw, Edentulous"[Mesh] OR "Tooth Extraction"[Mesh] OR "Alveolar Bone Loss"[Mesh] OR "Tooth Loss"[Mesh]) AND ("short* implant*" OR "short dental implant*" OR "extra?short dental implant*" OR "ultra?short dental implant*") AND (("Long* implant*" OR "Normal implant*" OR "standard implant*" OR "Conventional implant*" OR "Conventional length implant*" OR ("Dental Implants"[Mesh])) AND ("Bone Substitutes"[Mesh] OR "bone augment*" OR "bone regenerat*" OR "augmented bone" OR "ridge augmentation" OR "bone graft*"))	Year: 2012-2022	93
**SciELO**	((posterior mandible) OR (mandible) OR (posterior jaw) OR (inferior jaw) OR (partially edentulous jaw) OR (edentulous jaw) OR (Tooth Extraction) OR (Alveolar Bone Loss) OR (Tooth Loss) OR (mandíbula posterior) OR mandíbula OR (desdentamiento parcial) OR (desdentamiento total) OR desdentado OR desdentada)) AND ((shorter implant) OR (short dental implant) OR (extra short dental implant) OR (ultra short dental implant) OR (implante corto) OR (implante ultra corto) OR (implante dental extra corto)) AND (((Longer implant) OR (Normal implant) OR (standard implant) OR (Conventional implant) OR (Conventional length implant) OR (Dental Implant) OR (implante dental) OR (implante convencional) OR (implante normal) OR (implante de largo estándar)) AND ((Bone Substitutes) OR (sustituto óseo) OR (bone augmentation) OR (bone regeneration) OR (augmented bone) OR (ridge augmentation) OR (bone grafting) OR (aumento óseo) OR regeneración ósea)))	-	0
**Scopus**	TITLE-ABS-KEY("posterior mandible*" OR "mandible*" OR "posterior jaw*" OR "inferior jaw*" OR "partially edentulous jaw*" OR "Edentulous jaw*" OR "Tooth Extraction" OR "Alveolar Bone Loss" OR "Tooth Loss") AND TITLE-ABS-KEY("short* implant*" OR "short dental implant*" OR "extra?short dental implant*" OR "ultra?short dental implant*") AND (TITLE-ABS-KEY("Long* implant*" OR "Normal implant*" OR "standard implant*" OR "Conventional implant*" OR "Conventional length implant*" OR "Dental Implant*") AND TITLE-ABS-KEY("Bone Substitut*" OR	Year: 2012-2022	111

	"bone augment*" OR "bone regenerat*" OR "augmented bone" OR "ridge augmentation" OR "bone graft*"))		
**Web of Science**	#1: TS=("posterior mandible*" OR "mandible*" OR "posterior jaw*" OR "inferior jaw*" OR "partially edentulous jaw*" OR "edentulous jaw*" OR "Tooth Extraction" OR "Alveolar Bone Loss" OR "Tooth Loss")<br>#2: TS=("short* implant*" OR "short dental implant*" OR "extra?short dental implant*" OR "ultra?short dental implant*")<br>#3: TS=(("Long* implant*" OR "Normal implant*" OR "standard implant*" OR "Conventional implant*" OR "Conventional length implant*" OR "Dental Implant*") AND ("Bone Substitut*" OR "bone augment*" OR "bone regenerat*" OR "augmented bone" OR "ridge augmentation" OR "bone graft*"))<br>#1 AND #2 AND #3	Year: 2012-2022	107

Table S2. Characteristics of the excluded studies

Reference	P	I	C	O	Excluded from	
					Screening	Evidence Matrix
**Systematic reviews and Meta-analysis**						
Al-Johany SS, 2019 ¶ [1]	○	○	●	○	●	○
Atieh 2012 ¶ [2]	○	○	●	○	●	○
Bitinas 2021¶ [3]	●	○	○	○	●	○
Chen 2019 ¶ [4]	●	○	○	○	●	○
Fernandez 2022 ¶ [5]	○	○	●	○	●	○
Guida 2022 ¶ [6]	○	○	●	○	●	○
Hagi 2004 † [7]	○	○	●	○	●	○
Iezzi 2020 ¶ [8]	●	○	○	○	●	○
Kotsovilis 2009 † [9]	○	○	●	○	●	○
Lee 2014 ¶ [10]	●	○	○	○	●	○
Liang 2022 † [11]	Indirectly excluded from the "Evidence Matrix" (all primary studies included in this review were excluded)				○	●
Monje 2013 ¶ [12]	○	○	●	○	●	○
Moraschini 2021 ¶ [13]	○	○	●	○	●	○
Neldam 2012 † [14]	○	○	●	○	●	○
Papaspyridakos 2018 ¶ [15]	●	○	○	○	●	○
Pommer 2011 † [16]	○	○	●	○	●	○
Ravida 2019 † [17]	●	○	○	○	●	○
Rokn 2018 ¶ [18]	Protocol of Bitaraf 2019 [19]				●	○
Sun 2011 † [20]	○	○	●	○	●	○
Telleman 2011 † [21]	○	○	●	○	●	○
Tolentino da Roza de Souza 2018 † [22]	Indirectly excluded from the "Evidence Matrix" (all primary studies included in this review were excluded)				○	●
Uehara 2018 ¶ [23]	●	○	○	○	●	○
Vazouras 2020 ¶ [24]	○	○	●	○	●	○
**Primary studies**						
Amorfini 2014 [25]	○	○	●	○	○	●
Arlin 2006 [26]*	○	○	●	○	○	●

Baek 2019 [27]	○	○	●	○	●	○
Barausse 2022 [28]	○	○	●	○	●	○
Bechara 2017 [29]	●	○	○	○	○	●
Bianchi 2008 [30]	○	○	●	○	○	●
Bolle 2018 [31]	○	○	●	○	○	●
Cannizzaro 2009 [32]	●	○	○	○	○	●
Cannizzaro 2013 [33]	●	○	○	○	○	●
Cannizzaro 2015 [34]	○	○	●	○	○	●
Cannizzaro 2018 [35]	○	○	●	○	○	●
Chiapasco 2004 [36]	○	○	●	○	○	●
Chiapasco 2007 [37]	○	○	●	○	○	●
Chiapasco 2013 [38]	○	○	●	○	○	●
Cordaro 2011 [39]	○	○	●	○	○	●
de Molon 2017 [40]**	○	○	●	○	●	○
Dottore 2014 [41]	○	○	●	○	○	●
Esposito 2012 [42]	Preliminary results from thread Pistilli 2013 [43]; Felice 2018 [44], 2019 [45]				●	○
Esposito 2015 [46]	●	○	○	○	○	●
Esposito 2015 [47]	○	○	●	○	○	●
Esposito 2016 [48]	○	○	●	○	○	●
Felice 2009 [49]	○	○	●	○	○	●
Felice 2009 [50]	○	○	●	○	○	●
Felice 2012 [51]	Preliminary results from thread Pistilli 2013 [52], Gastaldi 2018 [53], Esposito 2019 [54]				●	○
Felice 2015 [55]	●	○	○	○	○	●
Felice 2016 [56]	○	○	●	○	○	●
Felice 2020 [57]	○	○	●	○	●	○
Ferrigno 2006 [58]	●	○	○	○	○	●
Fontana 2008 [59]	○	○	●	○	○	●
Gastaldi 2017 [60]	●	○	○	○	○	●
Guida 2020 [61]	○	○	●	○	○	●
Guljé 2013 [62]	○	○	●	○	○	●
Guljé 2014 [63]	●	○	○	○	○	●
Guljé 2019 [64]	●	○	○	○	○	●
Hadzik 2018 [65]	●	○	○	○	○	●
Karci 2021 [66]	○	○	●	○	●	○
Mendoza-Azpur 2016 [67]	○	○	●	○	○	●
Merli 2010 [68]	○	○	●	○	○	●
Merli 2014 [69]	○	○	●	○	○	●
Naenni 2018 [70]	○	○	●	○	○	●
Peñarrocha-Oltra 2014 [71]	○	○	●	○	●	○
Pohl 2017 [72]	●	○	○	○	○	●
Queiroz 2015 [73]	○	○	●	○	○	●
Rokn 2018 [74]	○	○	●	○	○	●
Romeo 2014 [75]	○	○	●	○	○	●
Ronda 2014 [76]	○	○	●	○	○	●
Rossi 2016 [77]	○	○	●	○	○	●
Sahrmann 2016 [78]	○	○	●	○	○	●

Schincaglia 2015 [79]	●	○	○	○	○	●
Shah 2018 [80]**	●	○	○	○	●	○
Shi 2019 [81]	●	○	○	○	○	●
Stellingsma 2014 [82]	○	○	●	○	○	●
Storelli 2018 [83]	○	○	●	○	○	●
Thoma 2015 [84]	●	○	○	○	○	●
Thoma 2018 [85]	●	○	○	○	○	●
Weerapong 2019 [86]	○	○	●	○	●	○
Yu 2017 [87]	●	○	○	○	○	●
Zadeh 2018 [88]	○	○	●	○	●	○

*Seek for information, with affirmative response, **The author does not respond to the data inquiry

References Table S2

1. Al-Johany SS. Survival Rates of Short Dental Implants (</= 6.5 mm) Placed in Posterior Edentulous Ridges and Factors Affecting their Survival after a 12-Month Follow-up Period: A Systematic Review. Int J Oral Maxillofac Implants. 2019;34(3):605-21.
2. Atieh MA, Zadeh H, Stanford CM, Cooper LF. Survival of short dental implants for treatment of posterior partial edentulism: a systematic review. Int J Oral Maxillofac Implants. 2012;27(6):1323-31.
3. Bitinas D, Bardijevskytė G. Short implants without bone augmentation versus long implants with bone augmentation. Systematic review and meta-analysis. Australian dental journal. 2021.
4. Chen S, Ou Q, Wang Y, Lin X. Short Implants (5-8 mm) Versus Long Implants (≥10 mm) with Augmentation in Atrophic Posterior Jaws: A Meta-Analysis of Randomized Controlled Trials. Journal of oral rehabilitation. 2019;46(12):1192-203.
5. Fernandes G, Costa B, Trindade HF, Castilho RM, Fernandes J. Comparative analysis between extra-short implants (</=6 mm) and 6 mm-longer implants: a meta-analysis of randomized controlled trial. Aust Dent J. 2022.
6. Guida L, Bressan E, Cecoro G, Volpe AD, Del Fabbro M, Annunziata M. Short versus Longer Implants in Sites without the Need for Bone Augmentation: A Systematic Review and Meta-Analysis of Randomized Controlled Trials. Materials (Basel). 2022;15(9).
7. Hagi D, Deporter DA, Pilliar RM, Arenovich T. A targeted review of study outcomes with short (< or = 7 mm) endosseous dental implants placed in partially edentulous patients. J Periodontol. 2004;75(6):798-804.
8. Iezzi G, Perrotti V, Felice P, Barausse C, Piattelli A, Del Fabbro M. Are <7-mm long implants in native bone as effective as longer implants in augmented bone for the rehabilitation of posterior atrophic jaws? A systematic review and meta-analysis. Clinical implant dentistry and related research. 2020.
9. Kotsovilis S, Fourmousis I, Karoussis IK, Bamia C. A systematic review and meta-analysis on the effect of implant length on the survival of rough-surface dental implants. J Periodontol. 2009;80(11):1700-18.
10. Lee SA, Lee CT, Fu MM, Elmisalati W, Chuang SK. Systematic review and meta-analysis of randomized controlled trials for the management of limited vertical height in the posterior region: short implants (5 to 8 mm) vs longer implants (> 8 mm) in vertically augmented sites. The International journal of oral & maxillofacial implants. 2014;29(5):1085-97.
11. Liang L, Wu X, Yan Q, Shi B. Are short implants (≤8.5 mm) reliable in the rehabilitation of completely edentulous patients: A systematic review and meta-analysis. The Journal of prosthetic dentistry. 2022.
12. Monje A, Chan HL, Fu JH, Suarez F, Galindo-Moreno P, Wang HL. Are short dental implants (<10 mm) effective? a meta-analysis on prospective clinical trials. J Periodontol. 2013;84(7):895-904.
13. Moraschini V, Mourao C, Montemezzi P, Kischinhevsky ICC, de Almeida DCF, Javid K, et al. Clinical Comparation of Extra-Short (4 mm) and Long (>8 mm) Dental Implants Placed in Mandibular Bone: A Systematic Review and Metanalysis. Healthcare (Basel). 2021;9(3).
14. Neldam CA, Pinholt EM. State of the art of short dental implants: a systematic review of the literature. Clin Implant Dent Relat Res. 2012;14(4):622-32.
15. Papaspyridakos P, De Souza A, Vazouras K, Gholami H, Pagni S, Weber HP. Survival rates of short dental implants (≤6 mm) compared with implants longer than 6 mm in posterior jaw areas: A meta-analysis. Clinical oral implants research. 2018;29 Suppl 16:8-20.
16. Pommer B, Frantal S, Willer J, Posch M, Watzek G, Tepper G. Impact of dental implant length on early failure rates: a meta-analysis of observational studies. J Clin Periodontol. 2011;38(9):856-63.

17. Ravida A, Wang IC, Sammartino G, Barootchi S, Tattan M, Troiano G, et al. Prosthetic Rehabilitation of the Posterior Atrophic Maxilla, Short (</=6 mm) or Long (>/=10 mm) Dental Implants? A Systematic Review, Meta-analysis, and Trial Sequential Analysis: Naples Consensus Report Working Group A. Implant Dent. 2019;28(6):590-602.
18. Rokn AR, Keshtkar A, Monzavi A, Hashemi K, Bitaraf T. Comparing Short Dental Implants to Standard Dental Implants: Protocol for a Systematic Review. JMIR Res Protoc. 2018;7(1):e16.
19. Bitaraf T, Keshtkar A, Rokn AR, Monzavi A, Geramy A, Hashemi K. Comparing short dental implant and standard dental implant in terms of marginal bone level changes: A systematic review and meta-analysis of randomized controlled trials. Clinical implant dentistry and related research. 2019;21(4):796-812.
20. Sun HL, Huang C, Wu YR, Shi B. Failure rates of short (≤ 10 mm) dental implants and factors influencing their failure: a systematic review. Int J Oral Maxillofac Implants. 2011;26(4):816-25.
21. Telleman G, Raghoebar GM, Vissink A, den Hartog L, Huddleston Slater JJ, Meijer HJ. A systematic review of the prognosis of short (<10 mm) dental implants placed in the partially edentulous patient. J Clin Periodontol. 2011;38(7):667-76.
22. Tolentino da Rosa de Souza P, Binhame Albini Martini M, Reis Azevedo-Alanis L. Do short implants have similar survival rates compared to standard implants in posterior single crown?: A systematic review and meta-analysis. Clin Implant Dent Relat Res. 2018;20(5):890-901.
23. Uehara PN, Matsubara VH, Igai F, Sesma N, Mukai MK, Araujo MG. Short Dental Implants (≤7mm) Versus Longer Implants in Augmented Bone Area: A Meta-Analysis of Randomized Controlled Trials. The open dentistry journal. 2018;12:354-65.
24. Vazouras K, de Souza AB, Gholami H, Papaspyridakos P, Pagni S, Weber HP. Effect of time in function on the predictability of short dental implants (</=6 mm): A meta-analysis. J Oral Rehabil. 2020;47(3):403-15.
25. Amorfini L, Migliorati M, Signori A, Silvestrini-Biavati A, Benedicenti S. Block allograft technique versus standard guided bone regeneration: a randomized clinical trial. Clin Implant Dent Relat Res. 2014;16(5):655-67.
26. Arlin ML. Short dental implants as a treatment option: results from an observational study in a single private practice. Int J Oral Maxillofac Implants. 2006;21(5):769-76.
27. Baek Y-W, Lim Y-J, Lee J, Koo K-T, Kim M-J, Kwon H-B. One-Year Results of a Randomized Controlled Clinical Trial of Immediately Loaded Short Implants Placed in the Lower Posterior Single Molar Using a Complete Digital Workflow. Applied Sciences. 2019;9(7).
28. Barausse C, Pistilli R, Canullo L, Bonifazi L, Ferri A, Felice P. A 5-year randomized controlled clinical trial comparing 4-mm ultrashort to longer implants placed in regenerated bone in the posterior atrophic jaw. Clin Implant Dent Relat Res. 2022;24(1):4-12.
29. Bechara S, Kubilius R, Veronesi G, Pires JT, Shibli JA, Mangano FG. Short (6-mm) dental implants versus sinus floor elevation and placement of longer (>/=10-mm) dental implants: a randomized controlled trial with a 3-year follow-up. Clin Oral Implants Res. 2017;28(9):1097-107.
30. Bianchi A, Felice P, Lizio G, Marchetti C. Alveolar distraction osteogenesis versus inlay bone grafting in posterior mandibular atrophy: a prospective study. Oral Surg Oral Med Oral Pathol Oral Radiol Endod. 2008;105(3):282-92.
31. Bolle C, Felice P, Barausse C, Pistilli V, Trullenque-Eriksson A, Esposito M. 4 mm long vs longer implants in augmented bone in posterior atrophic jaws: 1-year post-loading results from a multicentre randomised controlled trial. Eur J Oral Implantol. 2018;11(1):31-47.
32. Cannizzaro G, Felice P, Leone M, Viola P, Esposito M. Early loading of implants in the atrophic posterior maxilla: lateral sinus lift with autogenous bone and Bio-Oss versus crestal mini sinus lift and 8-mm hydroxyapatite-coated implants. A randomised controlled clinical trial. Eur J Oral Implantol. 2009;2(1):25-38.
33. Cannizzaro G, Felice P, Minciarelli AF, Leone M, Viola P, Esposito M. Early implant loading in the atrophic posterior maxilla: 1-stage lateral versus crestal sinus lift and 8 mm hydroxyapatite-coated implants. A 5-year randomised controlled trial. Eur J Oral Implantol. 2013;6(1):13-25.
34. Cannizzaro G, Felice P, Buti J, Leone M, Ferri V, Esposito M. Immediate loading of fixed cross-arch prostheses supported by flapless-placed supershort or long implants: 1-year results from a randomised controlled trial. Eur J Oral Implantol. 2015;8(1):27-36.
35. Cannizzaro G, Felice P, Ippolito DR, Velasco-Ortega E, Esposito M. Immediate loading of fixed cross-arch prostheses supported by flapless-placed 5 mm or 11.5 mm long implants: 5-year results from a randomised controlled trial. Eur J Oral Implantol. 2018;11(3):295-306.
36. Chiapasco M, Romeo E, Casentini P, Rimondini L. Alveolar distraction osteogenesis vs. vertical guided bone regeneration for the correction of vertically deficient edentulous ridges: a 1-3-year prospective study on humans. Clin Oral Implants Res. 2004;15(1):82-95.

37. Chiapasco M, Zaniboni M, Rimondini L. Autogenous onlay bone grafts vs. alveolar distraction osteogenesis for the correction of vertically deficient edentulous ridges: a 2-4-year prospective study on humans. Clin Oral Implants Res. 2007;18(4):432-40.
38. Chiapasco M, Autelitano L, Rabbiosi D, Zaniboni M. The role of pericranium grafts in the reduction of postoperative dehiscences and bone resorption after reconstruction of severely deficient edentulous ridges with autogenous onlay bone grafts. Clin Oral Implants Res. 2013;24(6):679-87.
39. Cordaro L, Torsello F, Morcavallo S, di Torresanto VM. Effect of bovine bone and collagen membranes on healing of mandibular bone blocks: a prospective randomized controlled study. Clin Oral Implants Res. 2011;22(10):1145-50.
40. de Molon RS, Lages FS, Rivera CP, de Souza Faloni AP, Margonar R, Queiroz TP. Evaluation of Short and Regular Implants after Prosthesis Placement in the Mandible: A Nonrandomized Controlled Clinical Trial. J Contemp Dent Pract. 2017;18(12):1122-9.
41. Dottore AM, Kawakami PY, Bechara K, Rodrigues JA, Cassoni A, Figueiredo LC, et al. Stability of implants placed in augmented posterior mandible after alveolar osteotomy using resorbable nonceramic hydroxyapatite or intraoral autogenous bone: 12-month follow-up. Clin Implant Dent Relat Res. 2014;16(3):330-6.
42. Esposito M, Cannizzaro G, Soardi E, Pistilli R, Piattelli M, Corvino V, et al. Posterior atrophic jaws rehabilitated with prostheses supported by 6 mm-long, 4 mm-wide implants or by longer implants in augmented bone. Preliminary results from a pilot randomised controlled trial. Eur J Oral Implantol. 2012;5(1):19-33.
43. Pistilli R, Felice P, Cannizzaro G, Piatelli M, Corvino V, Barausse C, et al. Posterior atrophic jaws rehabilitated with prostheses supported by 6 mm long 4 mm wide implants or by longer implants in augmented bone. One-year post-loading results from a pilot randomised controlled trial. European journal of oral implantology. 2013;6(4):359-72.
44. Felice P, Barausse C, Pistilli V, Piattelli M, Ippolito DR, Esposito M. Posterior atrophic jaws rehabilitated with prostheses supported by 6 mm long × 4 mm wide implants or by longer implants in augmented bone. 3-year post-loading results from a randomised controlled trial. European journal of oral implantology. 2018;11(2):175-87.
45. Felice P, Pistilli R, Barausse C, Piattelli M, Buti J, Esposito M. Posterior atrophic jaws rehabilitated with prostheses supported by 6-mm-long 4-mm-wide implants or by longer implants in augmented bone. Five-year post-loading results from a within-person randomised controlled trial. International journal of oral implantology (Berlin, Germany). 2019;12(1):57-72.
46. Esposito M, Barausse C, Pistilli R, Sammartino G, Grandi G, Felice P. Short implants versus bone augmentation for placing longer implants in atrophic maxillae: One-year post-loading results of a pilot randomised controlled trial. Eur J Oral Implantol. 2015;8:257-68.
47. Esposito M, Barausse C, Pistilli R, Checchi V, Diazzi M, Gatto MR, et al. Posterior jaws rehabilitated with partial prostheses supported by 4.0 x 4.0 mm or by longer implants: Four-month post-loading data from a randomised controlled trial. European journal of oral implantology. 2015;8(3):221-30.
48. Esposito M, Zucchelli G, Barausse C, Pistilli R, Trullenque-Eriksson A, Felice P. Four mm-long versus longer implants in augmented bone in atrophic posterior jaws: 4-month post-loading results from a multicentre randomised controlled trial. European journal of oral implantology. 2016;9(4):393-409.
49. Felice P, Pistilli R, Lizio G, Pellegrino G, Nisii A, Marchetti C. Inlay versus onlay iliac bone grafting in atrophic posterior mandible: a prospective controlled clinical trial for the comparison of two techniques. Clin Implant Dent Relat Res. 2009;11 Suppl 1:e69-82.
50. Felice P, Marchetti C, Iezzi G, Piattelli A, Worthington H, Pellegrino G, et al. Vertical ridge augmentation of the atrophic posterior mandible with interpositional bloc grafts: bone from the iliac crest vs. bovine anorganic bone. Clinical and histological results up to one year after loading from a randomized-controlled clinical trial. Clin Oral Implants Res. 2009;20(12):1386-93.
51. Felice P, Pistilli R, Piattelli M, Soardi E, Corvino V, Esposito M. Posterior atrophic jaws rehabilitated with prostheses supported by 5 x 5 mm implants with a novel nanostructured calcium-incorporated titanium surface or by longer implants in augmented bone. Preliminary results from a randomised controlled trial. Eur J Oral Implantol. 2012;5(2):149-61.
52. Pistilli R, Felice P, Piattelli M, Gessaroli M, Soardi E, Barausse C, et al. Posterior atrophic jaws rehabilitated with prostheses supported by 5 x 5 mm implants with a novel nanostructured calcium-incorporated titanium surface or by longer implants in augmented bone. One-year results from a randomised controlled trial. European journal of oral implantology. 2013;6(4):343-57.
53. Gastaldi G, Felice P, Pistilli V, Barausse C, Ippolito DR, Esposito M. Posterior atrophic jaws rehabilitated with prostheses supported by 5 × 5 mm implants with a nanostructured calcium-incorporated titanium surface or by

longer implants in augmented bone. 3-year results from a randomised controlled trial. European journal of oral implantology. 2018;11(1):49-61.
54. Esposito M, Barausse C, Pistilli R, Piattelli M, Di Simone S, Ippolito DR, et al. Posterior atrophic jaws rehabilitated with prostheses supported by 5 × 5 mm implants with a nanostructured calcium-incorporated titanium surface or by longer implants in augmented bone. Five-year results from a randomised controlled trial. International journal of oral implantology (Berlin, Germany). 2019;12(1):39-54.
55. Felice P, Pistilli R, Barausse C, Bruno V, Trullenque-Eriksson A, Esposito M. Short implants as an alternative to crestal sinus lift: A 1-year multicentre randomised controlled trial. Eur J Oral Implantol. 2015;8(4):375-84.
56. Felice P, Checchi L, Barausse C, Pistilli R, Sammartino G, Masi I, et al. Posterior jaws rehabilitated with partial prostheses supported by 4.0 x 4.0 mm or by longer implants: One-year post-loading results from a multicenter randomised controlled trial. Eur J Oral Implantol. 2016;9(1):35-45.
57. Felice P, Barausse C, Pistilli R, Kalemaj Z, Esposito M. Four-mm-long versus longer implants in augmented bone in atrophic posterior jaws: three-year post-loading results from a multicentre randomised controlled trial. Clinical Trials in Dentistry 2020;02(1):5-26.
58. Ferrigno N, Laureti M, Fanali S. Dental implants placement in conjunction with osteotome sinus floor elevation: a 12-year life-table analysis from a prospective study on 588 ITI implants. Clin Oral Implants Res. 2006;17(2):194-205.
59. Fontana F, Santoro F, Maiorana C, Iezzi G, Piattelli A, Simion M. Clinical and histologic evaluation of allogeneic bone matrix versus autogenous bone chips associated with titanium-reinforced e-PTFE membrane for vertical ridge augmentation: a prospective pilot study. Int J Oral Maxillofac Implants. 2008;23(6):1003-12.
60. Gastaldi G, Felice P, Pistilli R, Barausse C, Trullenque-Eriksson A, Esposito M. Short implants as an alternative to crestal sinus lift: a 3-year multicentre randomised controlled tria. Eur J Oral Implantol. 2017;10(4):391-400.
61. Guida L, Annunziata M, Esposito U, Sirignano M, Torrisi P, Cecchinato D. 6-mm-short and 11-mm-long implants compared in the full-arch rehabilitation of the edentulous mandible: A 3-year multicenter randomized controlled trial. Clin Oral Implants Res. 2020;31(1):64-73.
62. Gulje F, Abrahamsson I, Chen S, Stanford C, Zadeh H, Palmer R. Implants of 6 mm vs. 11 mm lengths in the posterior maxilla and mandible: a 1-year multicenter randomized controlled trial. Clin Oral Implants Res. 2013;24(12):1325-31.
63. Guljé FL, Raghoebar GM, Vissink A, Meijer HJ. Single crowns in the resorbed posterior maxilla supported by either 6-mm implants or by 11-mm implants combined with sinus floor elevation surgery: a 1-year randomised controlled trial. Eur J Oral Implantol. 2014;7(3):247-55.
64. Guljé FL, Raghoebar GM, Vissink A, Meijer H. Single crowns in the resorbed posterior maxilla supported by either 11-mm implants combined with sinus floor elevation or 6-mm implants:A 5-year randomised controlled trial. Int J Oral Implantol (Berl). 2019;12(3):315-26.
65. Hadzik J, Krawiec M, Kubasiewicz-Ross P, Prylinska-Czyzewska A, Gedrange T, Dominiak M. Short Implants and Conventional Implants in The Residual Maxillary Alveolar Ridge: A 36-Month Follow-Up Observation. Med Sci Monit. 2018;24:5645-52.
66. Karci BL, Oncu E. Comparison of Osteoimmunological and Microbiological Parameters of Extra Short and Longer Implants Loaded in the Posterior Mandible: A Split Mouth Randomized Clinical Study. Acta Stomatol Croat. 2021;55(3):238-47.
67. Mendoza-Azpur G, Lau M, Valdivia E, Rojas J, Munoz H, Nevins M. Assessment of Marginal Peri-implant Bone-Level Short-Length Implants Compared with Standard Implants Supporting Single Crowns in a Controlled Clinical Trial: 12-Month Follow-up. Int J Periodontics Restorative Dent. 2016;36(6):791-5.
68. Merli M, Lombardini F, Esposito M. Vertical ridge augmentation with autogenous bone grafts 3 years after loading: resorbable barriers versus titanium-reinforced barriers. A randomized controlled clinical trial. Int J Oral Maxillofac Implants. 2010;25(4):801-7.
69. Merli M, Moscatelli M, Mariotti G, Rotundo R, Bernardelli F, Nieri M. Bone level variation after vertical ridge augmentation: resorbable barriers versus titanium-reinforced barriers. A 6-year double-blind randomized clinical trial. Int J Oral Maxillofac Implants. 2014;29(4):905-13.
70. Naenni N, Sahrmann P, Schmidlin PR, Attin T, Wiedemeier DB, Sapata V, et al. Five-Year Survival of Short Single-Tooth Implants (6 mm): A Randomized Controlled Clinical Trial. J Dent Res. 2018;97(8):887-92.
71. Penarrocha-Oltra D, Aloy-Prosper A, Cervera-Ballester J, Penarrocha-Diago M, Canullo L, Penarrocha-Diago M. Implant treatment in atrophic posterior mandibles: vertical regeneration with block bone grafts versus implants with 5.5-mm intrabony length. Int J Oral Maxillofac Implants. 2014;29(3):659-66.

72. Pohl V, Thoma DS, Sporniak-Tutak K, Garcia-Garcia A, Taylor TD, Haas R, et al. Short dental implants (6 mm) versus long dental implants (11-15 mm) in combination with sinus floor elevation procedures: 3-year results from a multicentre, randomized, controlled clinical trial. J Clin Periodontol. 2017;44(4):438-45.
73. Queiroz TP, Aguiar SC, Margonar R, de Souza Faloni AP, Gruber R, Luvizuto ER. Clinical study on survival rate of short implants placed in the posterior mandibular region: resonance frequency analysis. Clin Oral Implants Res. 2015;26(9):1036-42.
74. Rokn AR, Monzavi A, Panjnoush M, Hashemi HM, Kharazifard MJ, Bitaraf T. Comparing 4-mm dental implants to longer implants placed in augmented bones in the atrophic posterior mandibles: One-year results of a randomized controlled trial. Clinical implant dentistry and related research. 2018;20(6):997-1002.
75. Romeo E, Storelli S, Casano G, Scanferla M, Botticelli D. Six-mm versus 10-mm long implants in the rehabilitation of posterior edentulous jaws: a 5-year follow-up of a randomised controlled trial. Eur J Oral Implantol. 2014;7(4):371-81.
76. Ronda M, Rebaudi A, Torelli L, Stacchi C. Expanded vs. dense polytetrafluoroethylene membranes in vertical ridge augmentation around dental implants: a prospective randomized controlled clinical trial. Clin Oral Implants Res. 2014;25(7):859-66.
77. Rossi F, Botticelli D, Cesaretti G, De Santis E, Storelli S, Lang NP. Use of short implants (6 mm) in a single-tooth replacement: a 5-year follow-up prospective randomized controlled multicenter clinical study. Clin Oral Implants Res. 2016;27(4):458-64.
78. Sahrmann P, Naenni N, Jung RE, Held U, Truninger T, Hammerle CH, et al. Success of 6-mm Implants with Single-Tooth Restorations: A 3-year Randomized Controlled Clinical Trial. J Dent Res. 2016;95(6):623-8.
79. Schincaglia GP, Thoma DS, Haas R, Tutak M, Garcia A, Taylor TD, et al. Randomized controlled multicenter study comparing short dental implants (6 mm) versus longer dental implants (11-15 mm) in combination with sinus floor elevation procedures. Part 2: clinical and radiographic outcomes at 1 year of loading. J Clin Periodontol. 2015;42(11):1042-51.
80. Shah SN, Chung J, Kim DM, Machtei EE. Can extra-short dental implants serve as alternatives to bone augmentation? A preliminary longitudinal randomized controlled clinical trial. Quintessence international (Berlin, Germany : 1985). 2018;49(8):635-43.
81. Shi JY, Li Y, Qiao SC, Gu YX, Xiong YY, Lai HC. Short versus longer implants with osteotome sinus floor elevation for moderately atrophic posterior maxillae: A 1-year randomized clinical trial. J Clin Periodontol. 2019;46(8):855-62.
82. Stellingsma K, Raghoebar GM, Visser A, Vissink A, Meijer HJ. The extremely resorbed mandible, 10-year results of a randomized controlled trial on 3 treatment strategies. Clinical oral implants research. 2014;25(8):926-32.
83. Storelli S, Abbà A, Scanferla M, Botticelli D, Romeo E. 6 mm vs 10 mm-long implants in the rehabilitation of posterior jaws: A 10-year follow-up of a randomised controlled trial. Eur J Oral Implantol. 2018;11(3):283-92.
84. Thoma DS, Haas R, Tutak M, Garcia A, Schincaglia GP, Hammerle CH. Randomized controlled multicentre study comparing short dental implants (6 mm) versus longer dental implants (11-15 mm) in combination with sinus floor elevation procedures. Part 1: demographics and patient-reported outcomes at 1 year of loading. J Clin Periodontol. 2015;42(1):72-80.
85. Thoma DS, Haas R, Sporniak-Tutak K, Garcia A, Taylor TD, Hammerle CHF. Randomized controlled multicentre study comparing short dental implants (6 mm) versus longer dental implants (11-15 mm) in combination with sinus floor elevation procedures: 5-Year data. J Clin Periodontol. 2018;45(12):1465-74.
86. Weerapong K, Sirimongkolwattana S, Sastraruji T, Khongkhunthian P. Comparative study of immediate loading on short dental implants and conventional dental implants in the posterior mandible: A randomized clinical trial. Int J Oral Maxillofac Implants. 2019;34(1):141-9.
87. Yu H, Wang X, Qiu L. Outcomes of 6.5-mm Hydrophilic Implants and Long Implants Placed with Lateral Sinus Floor Elevation in the Atrophic Posterior Maxilla: A Prospective, Randomized Controlled Clinical Comparison. Clin Implant Dent Relat Res. 2017;19(1):111-22.
88. Zadeh HH, Gulje F, Palmer PJ, Abrahamsson I, Chen S, Mahallati R, et al. Marginal bone level and survival of short and standard-length implants after 3 years: An Open Multi-Center Randomized Controlled Clinical Trial. Clin Oral Implants Res. 2018;29(8):894-906.

Reference	Short implants Events	Short implants Total	Regular implants Events	Regular implants Total	Weight	Risk Ratio M-H, Random, 95% CI	Raw meta-analytic model Weight
**a. Implant Failure (before and after loading) at 1 year of follow-up**							
**a.1. Short implant length: 4 to <6 mm**							
Esposito 2011	0	26	1	30	6.4%	0.38 [0.02, 9.01]	2.4%
Pistilli 2013	0	32	2	31	7.2%	0.19 [0.01, 3.88]	2.6%
**Subtotal (95% CI)**		**58**	**61**		**13.6%**	**0.27 [0.03, 2.35]**	
Total events	0		3				
Heterogeneity: Tau² = 0.00; Chi² = 0.09, df = 1 (P = 0.76); I² = 0%							
Test for overall effect: Z = 1.19 (P = 0.23)							
**a.2. Short implant length: 6 to 8.5 mm**							
Bernardi 2018	5	86	13	84	66.1%	0.38 [0.14, 1.01]	24.3%
Felice 2010	1	60	3	61	12.9%	0.34 [0.04, 3.17]	4.7%
Pistilli 2013	0	41	3	47	7.5%	0.16 [0.01, 3.07]	2.7%
**Subtotal (95% CI)**		**187**		**192**	**86.4%**	**0.34 [0.15, 0.82]**	
Total events	6		19				
Heterogeneity: Tau² = 0.00; Chi² = 0.28, df = 2 (P = 0.87); I² = 0%							
Test for overall effect: Z = 2.42 (P = 0.02)							
**Total (95% CI)**		**245**		**253**	**100.0%**	**0.33 [0.15, 0.74]**	
Total events	6		22				
Heterogeneity: Tau² = 0.00; Chi² = 0.42, df = 4 (P = 0.98); I² = 0%							
Test for overall effect: Z = 2.69 (P = 0.007)							
Test for subgroup differences: Chi² = 0.04, df = 1 (P = 0.83), I² = 0%							
**b. Implant Failure (before and after loading) at 3 years of follow-up**							
**b.1. Short implant length: 4 to <6 mm**							
Esposito 2014	2	26	1	30	22.6%	2.31 [0.22, 24.01]	4.3%
Gastaldi 2018	1	32	2	31	22.5%	0.48 [0.05, 5.07]	4.3%
**Subtotal (95% CI)**		**58**		**61**	**45.2%**	**1.06 [0.20, 5.57]**	
Total events	3		3				
Heterogeneity: Tau² = 0.00; Chi² = 0.85, df = 1 (P = 0.36); I² = 0%							
Test for overall effect: Z = 0.07 (P = 0.95)							
**b.2. Short implant length: 6 to 8.5 mm**							
Esposito 2011	2	60	3	61	40.4%	0.68 [0.12, 3.91]	7.7%
Felice 2018	0	41	3	47	14.4%	0.16 [0.01, 3.07]	2.7%
**Subtotal (95% CI)**		**101**		**108**	**54.8%**	**0.47 [0.10, 2.10]**	
Total events	2		6				
Heterogeneity: Tau² = 0.00; Chi² = 0.70, df = 1 (P = 0.40); I² = 0%							
Test for overall effect: Z = 0.99 (P = 0.32)							
**Total (95% CI)**		**159**		**169**	**100.0%**	**0.68 [0.22, 2.06]**	
Total events	5		9				
Heterogeneity: Tau² = 0.00; Chi² = 2.06, df = 3 (P = 0.56); I² = 0%							
Test for overall effect: Z = 0.69 (P = 0.49)							
Test for subgroup differences: Chi² = 0.52, df = 1 (P = 0.47), I² = 0%							
**c. Implant Failure (before and after loading) at 5 years of follow-up**							
**c.1. Short implant length: 4 to <6 mm**							
Esposito 2019	1	32	2	31	13.5%	0.48 [0.05, 5.07]	4.3%
Felice 2019	2	26	1	30	13.5%	2.31 [0.22, 24.01]	4.3%
**Subtotal (95% CI)**		**58**		**61**	**27.0%**	**1.06 [0.20, 5.57]**	
Total events	3		3				
Heterogeneity: Tau² = 0.00; Chi² = 0.85, df = 1 (P = 0.36); I² = 0%							
Test for overall effect: Z = 0.07 (P = 0.95)							
**c.2. Short implant length: 6 to 8.5 mm**							
Felice 2019	2	41	3	47	24.5%	0.76 [0.13, 4.35]	7.8%
Felice 2014	5	60	3	61	38.6%	1.69 [0.42, 6.78]	12.3%
Pieri 2017	1	46	1	51	9.9%	1.11 [0.07, 17.22]	3.1%
**Subtotal (95% CI)**		**147**		**159**	**73.0%**	**1.22 [0.45, 3.36]**	
Total events	8		7				
Heterogeneity: Tau² = 0.00; Chi² = 0.50, df = 2 (P = 0.78); I² = 0%							
Test for overall effect: Z = 0.39 (P = 0.69)							
**Total (95% CI)**		**205**		**220**	**100.0%**	**1.18 [0.50, 2.79]**	
Total events	11		10				
Heterogeneity: Tau² = 0.00; Chi² = 1.37, df = 4 (P = 0.85); I² = 0%							
Test for overall effect: Z = 0.37 (P = 0.71)							
Test for subgroup differences: Chi² = 0.02, df = 1 (P = 0.88), I² = 0%							
**d. Implant Failure (before and after loading) at 8 years of follow-up**							
**d.1. Short implant length: 6 to 8.5 mm**							
Felice 2018	5	60	3	61	100.0%	1.69 [0.42, 6.78]	12.3%
**Subtotal (95% CI)**		**60**		**61**	**100.0%**	**1.69 [0.42, 6.78]**	
Total events	5		3				
Heterogeneity: Not applicable							
Test for overall effect: Z = 0.75 (P = 0.46)							
**Total (95% CI)**		**60**		**61**	**100.0%**	**1.69 [0.42, 6.78]**	
Total events	5		3				
Heterogeneity: Not applicable							
Test for overall effect: Z = 0.75 (P = 0.46)							
Test for subgroup differences: Not applicable							
**Total (95% CI)**		**669**		**703**	**100.0%**	**0.70 [0.43, 1.13]**	
Total events	27		44				
Heterogeneity: Tau² = 0.00; Chi² = 10.15, df = 14 (P = 0.75); I² = 0%							
Test for overall effect: Z = 1.46 (P = 0.14)							
Test for subgroup differences (follow-up): Chi² = 6.27, df = 3 (P = 0.10), I² = 52.2%							
Test for subgroup differences (short implant length): Chi² = 0.06, df = 1 (P = 0.81), I² = 0%							

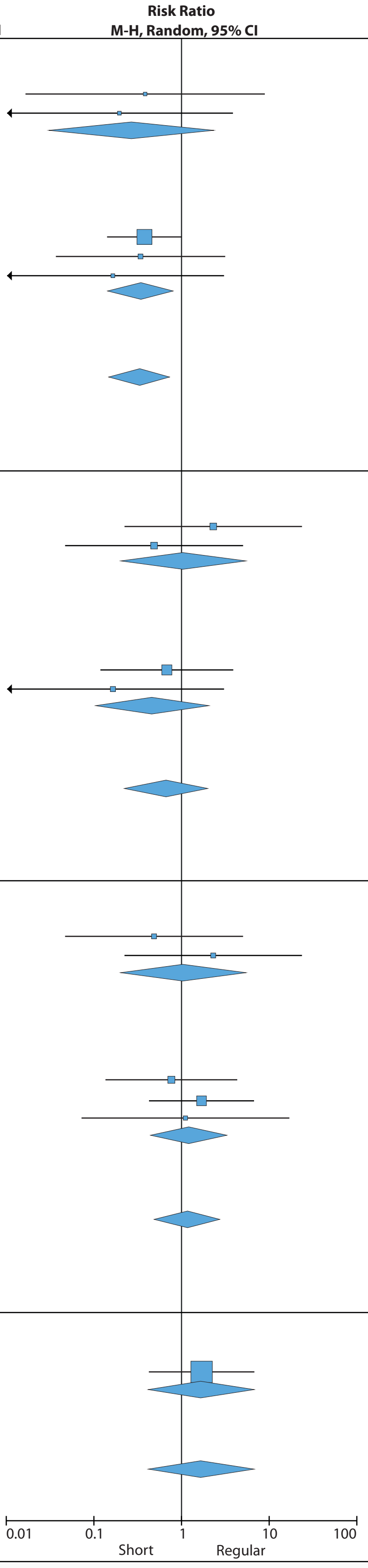

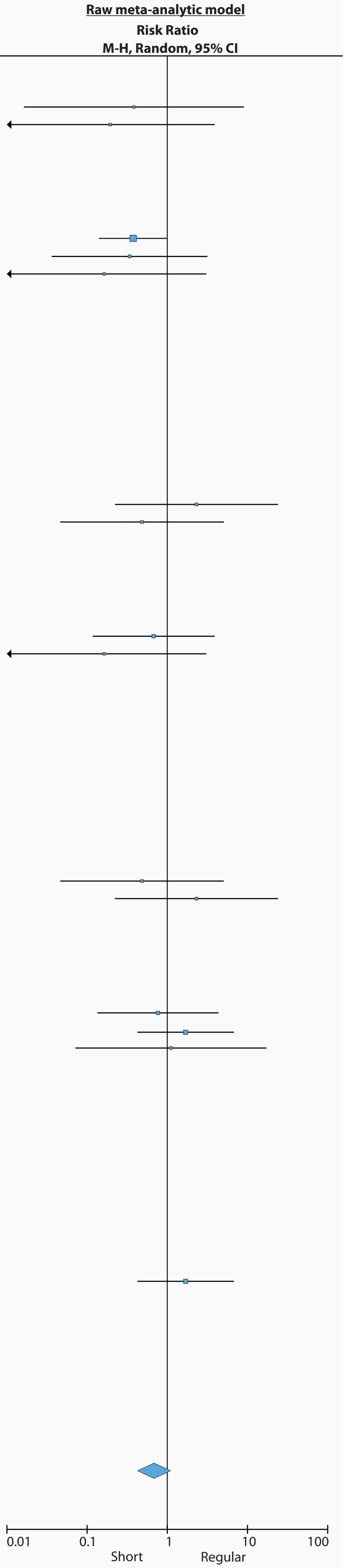

Fig. S1. Forest plot for the random effects meta-analysis assessing the effect of Short implants compared to Regular implants after Bone Augmentation for the posterior athropic mandible on Implant failure. CI: confidence interval, M-H: Mantel-Haenszel, SD: standard deviation

Reference	Short implants Mean [mm]	Short implants SD [mm]	Short implants Total	Regular implants Mean [mm]	Regular implants SD [mm]	Regular implants Total	Weight	Mean Difference IV, Random, 95% CI	Raw meta-analytic model Weight
**a. Marginal Bone Loss (change from baseline) at 1 year of follow-up**									
**a.1. Short implant length: 4 to <6 mm**									
Esposito 2011	1.2	0.49	15	1.2	0.47	15	4.2%	0.00 [-0.34, 0.34]	5.4%
Pistilli 2013	0.94	0.05	20	1.03	0.07	18	38.7%	-0.09 [-0.13, -0.05]	11.9%
**Subtotal (95% CI)**			**35**			**33**	**42.9%**	**-0.09 [-0.13, -0.05]**	
Heterogeneity: $Tau^2 = 0.00$; $Chi^2 = 0.26$, df = 1 (P = 0.61); $I^2 = 0\%$									
Test for overall effect: Z = 4.49 (P < 0.00001)									
**a.2. Short implant length: 6 to 8.5 mm**									
Felice 2010	1	0.36	29	1	0.31	30	13.0%	0.00 [-0.17, 0.17]	9.3%
Pieri 2017	0.3	0.24	23	0.75	0.68	22	5.3%	-0.45 [-0.75, -0.15]	6.3%
Pistilli 2013	1.05	0.06	19	1.07	0.06	18	38.8%	-0.02 [-0.06, 0.02]	11.9%
**Subtotal (95% CI)**			**71**			**70**	**57.1%**	**-0.10 [-0.28, 0.08]**	
Heterogeneity: $Tau^2 = 0.02$; $Chi^2 = 7.82$, df = 2 (P = 0.02); $I^2 = 74\%$									
Test for overall effect: Z = 1.10 (P = 0.27)									
**Total (95% CI)**			**106**			**103**	**100.0%**	**-0.07 [-0.14, 0.01]**	
Heterogeneity: $Tau^2 = 0.00$; $Chi^2 = 13.35$, df = 4 (P = 0.010); $I^2 = 70\%$									
Test for overall effect: Z = 1.76 (P = 0.08)									
Test for subgroup differences: $Chi^2 = 0.02$, df = 1 (P = 0.90), $I^2 = 0\%$									
**b. Marginal Bone Loss (change from baseline) at 3 year of follow-up**									
**b.1. Short implant length: 4 to <6 mm**									
Esposito 2014	1.44	0.44	13	1.63	0.52	14	12.0%	-0.19 [-0.55, 0.17]	5.1%
Gastaldi 2018	1.1	0.25	17	1.39	0.4	14	27.3%	-0.29 [-0.53, -0.05]	7.6%
**Subtotal (95% CI)**			**30**			**28**	**39.3%**	**-0.26 [-0.46, -0.06]**	
Heterogeneity: $Tau^2 = 0.00$; $Chi^2 = 0.20$, df = 1 (P = 0.65); $I^2 = 0\%$									
Test for overall effect: Z = 2.53 (P = 0.01)									
**b.2. Short implant length: 6 to 8.5 mm**									
Esposito 2011	1.24	0.36	27	1.76	0.72	28	17.7%	-0.52 [-0.82, -0.22]	6.3%
Felice 2018	1.25	0.35	15	1.54	0.14	14	43.0%	-0.29 [-0.48, -0.10]	8.8%
**Subtotal (95% CI)**			**42**			**42**	**60.7%**	**-0.38 [-0.59, -0.16]**	
Heterogeneity: $Tau^2 = 0.01$; $Chi^2 = 1.61$, df = 1 (P = 0.20); $I^2 = 38\%$									
Test for overall effect: Z = 3.38 (P = 0.0007)									
**Total (95% CI)**			**72**			**70**	**100.0%**	**-0.32 [-0.44, -0.19]**	
Heterogeneity: $Tau^2 = 0.00$; $Chi^2 = 2.36$, df = 3 (P = 0.50); $I^2 = 0\%$									
Test for overall effect: Z = 4.96 (P < 0.00001)									
Test for subgroup differences: $Chi^2 = 0.59$, df = 1 (P = 0.44), $I^2 = 0\%$									
**c. Marginal Bone Loss (change from baseline) at 5 year of follow-up**									
**c.1. Short implant length: 4 to <6 mm**									
Esposito 2019	1.22	0.27	14	1.7	0.46	10	28.4%	-0.48 [-0.80, -0.16]	5.9%
Felice 2019	1.72	0.57	10	2.1	0.61	10	12.7%	-0.38 [-0.90, 0.14]	3.2%
**Subtotal (95% CI)**			**24**			**20**	**41.1%**	**-0.45 [-0.72, -0.18]**	
Heterogeneity: $Tau^2 = 0.00$; $Chi^2 = 0.10$, df = 1 (P = 0.75); $I^2 = 0\%$									
Test for overall effect: Z = 3.27 (P = 0.001)									
**c.2. Short implant length: 6 to 8.5 mm**									
Felice 2019	1.34	0.35	12	2.11	0.59	12	20.8%	-0.77 [-1.16, -0.38]	4.7%
Felice 2014	1.49	0.4	25	2.34	0.75	25	26.5%	-0.85 [-1.18, -0.52]	5.6%
Pieri 2017	0.68	0.68	23	1.61	1.12	22	11.6%	-0.93 [-1.47, -0.39]	3.0%
**Subtotal (95% CI)**			**60**			**59**	**58.9%**	**-0.84 [-1.07, -0.61]**	
Heterogeneity: $Tau^2 = 0.00$; $Chi^2 = 0.23$, df = 2 (P = 0.89); $I^2 = 0\%$									
Test for overall effect: Z = 7.15 (P < 0.00001)									
**Total (95% CI)**			**84**			**79**	**100.0%**	**-0.68 [-0.87, -0.48]**	
Heterogeneity: $Tau^2 = 0.01$; $Chi^2 = 4.82$, df = 4 (P = 0.31); $I^2 = 17\%$									
Test for overall effect: Z = 6.80 (P < 0.00001)									
Test for subgroup differences: $Chi^2 = 4.49$, df = 1 (P = 0.03), $I^2 = 77.7\%$									
**d. Marginal Bone Loss (change from baseline) at 8 year of follow-up**									
**d.1. Short implant length: 6 to 8.5 mm**									
Felice 2018	1.58	0.46	24	2.46	0.8	23	100.0%	-0.88 [-1.26, -0.50]	4.9%
**Subtotal (95% CI)**			**24**			**23**	**100.0%**	**-0.88 [-1.26, -0.50]**	
Heterogeneity: Not applicable									
Test for overall effect: Z = 4.60 (P < 0.00001)									
**Total (95% CI)**			**24**			**23**	**100.0%**	**-0.88 [-1.26, -0.50]**	
Heterogeneity: Not applicable									
Test for overall effect: Z = 4.60 (P < 0.00001)									
Test for subgroup differences: Not applicable									
**Total (95% CI)**			**286**			**275**	**100.0%**	**-0.33 [-0.43, -0.22]**	
Heterogeneity: $Tau^2 = 0.03$; $Chi^2 = 99.25$, df = 14 (P < 0.00001); $I^2 = 86\%$									
Test for overall effect: Z = 5.93 (P < 0.00001)									
Test for subgroup differences (follow-up): $Chi^2 = 51.51$, df = 3 (P < 0.00001), $I^2 = 94.2\%$									
Test for subgroup differences (short implant length): $Chi^2 = 1.47$, df = 1 (P = 0.22), $I^2 = 32.2\%$									

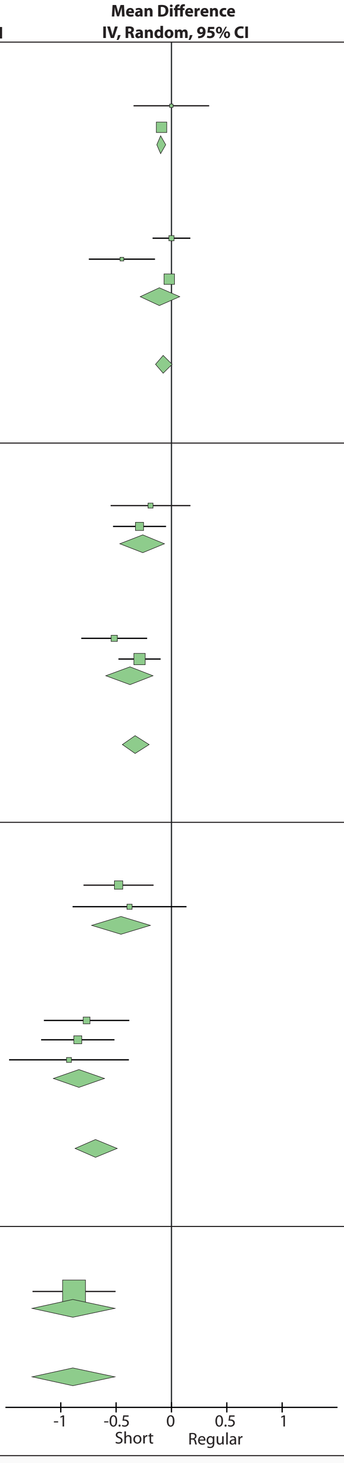

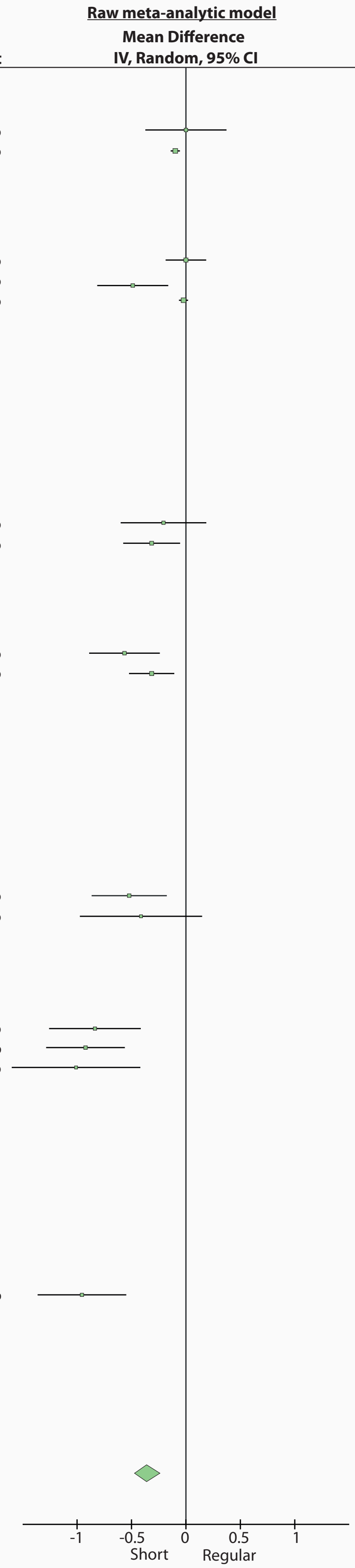

Fig. S2. Forest plot for the random effects meta-analysis assessing the effect of Short implants compared to Regular implants after Bone Augmentation for the posterior athropic mandible on Marginal bone loss. CI: confidence interval, IV: inverse variance, SD: standard deviation

Reference	Short implants Events	Short implants Total	Regular implants Events	Regular implants Total	Weight	Risk Ratio M-H, Random, 95% CI	Raw meta-analytic model Weight
**a. Biological complications (before and after loading) at 1 year of follow-up**							
**a.1. Short implant length: 4 to <6 mm**							
Esposito 2011	1	26	1	30	10.3%	1.15 [0.08, 17.54]	1.7%
Pistilli 2013	8	32	17	31	41.1%	0.46 [0.23, 0.90]	9.5%
**Subtotal (95% CI)**		**58**		**61**	**51.3%**	**0.48 [0.25, 0.93]**	
Total events	9		18				
Heterogeneity: Tau² = 0.00; Chi² = 0.42, df = 1 (P = 0.51); I² = 0%							
Test for overall effect: Z = 2.17 (P = 0.03)							
**a.2. Short implant length: 6 to 8.5 mm**							
Bernardi 2018	3	86	22	84	29.6%	0.13 [0.04, 0.43]	5.9%
Felice 2010	0	60	4	61	9.3%	0.11 [0.01, 2.05]	1.5%
Pistilli 2013	0	41	10	47	9.8%	0.05 [0.00, 0.90]	1.6%
**Subtotal (95% CI)**		**187**		**192**	**48.7%**	**0.12 [0.04, 0.32]**	
Total events	3		36				
Heterogeneity: Tau² = 0.00; Chi² = 0.35, df = 2 (P = 0.84); I² = 0%							
Test for overall effect: Z = 4.17 (P < 0.0001)							
**Total (95% CI)**		**245**		**253**	**100.0%**	**0.25 [0.09, 0.66]**	
Total events	12		54				
Heterogeneity: Tau² = 0.48; Chi² = 7.11, df = 4 (P = 0.13); I² = 44%							
Test for overall effect: Z = 2.79 (P = 0.005)							
Test for subgroup differences: Chi² = 5.33, df = 1 (P = 0.02), I² = 81.2%							
**b. Biological complications (before and after loading) at 3 years of follow-up**							
**b.1. Short implant length: 4 to <6 mm**							
Esposito 2014	9	26	12	30	29.7%	0.87 [0.44, 1.72]	9.4%
Gastaldi 2018	9	32	17	31	30.9%	0.51 [0.27, 0.97]	9.8%
**Subtotal (95% CI)**		**58**		**61**	**60.6%**	**0.66 [0.39, 1.09]**	
Total events	18		29				
Heterogeneity: Tau² = 0.02; Chi² = 1.20, df = 1 (P = 0.27); I² = 16%							
Test for overall effect: Z = 1.61 (P = 0.11)							
**b.2. Short implant length: 6 to 8.5 mm**							
Esposito 2011	5	60	22	61	24.4%	0.23 [0.09, 0.57]	7.6%
Felice 2018	2	41	13	47	15.0%	0.18 [0.04, 0.74]	4.6%
**Subtotal (95% CI)**		**101**		**108**	**39.4%**	**0.21 [0.10, 0.46]**	
Total events	7		35				
Heterogeneity: Tau² = 0.00; Chi² = 0.10, df = 1 (P = 0.75); I² = 0%							
Test for overall effect: Z = 3.96 (P < 0.0001)							
**Total (95% CI)**		**159**		**169**	**100.0%**	**0.42 [0.21, 0.84]**	
Total events	25		64				
Heterogeneity: Tau² = 0.29; Chi² = 7.87, df = 3 (P = 0.05); I² = 62%							
Test for overall effect: Z = 2.47 (P = 0.01)							
Test for subgroup differences: Chi² = 5.71, df = 1 (P = 0.02), I² = 82.5%							
**c. Biological complications (before and after loading) at 5 years of follow-up**							
**c.1. Short implant length: 4 to <6 mm**							
Esposito 2019	10	32	17	31	24.3%	0.57 [0.31, 1.04]	10.1%
Felice 2019	11	26	12	30	24.0%	1.06 [0.56, 1.98]	9.9%
**Subtotal (95% CI)**		**58**		**61**	**48.3%**	**0.77 [0.42, 1.42]**	
Total events	21		29				
Heterogeneity: Tau² = 0.09; Chi² = 1.94, df = 1 (P = 0.16); I² = 48%							
Test for overall effect: Z = 0.84 (P = 0.40)							
**c.2. Short implant length: 6 to 8.5 mm**							
Felice 2019	3	41	14	47	16.6%	0.25 [0.08, 0.80]	5.9%
Felice 2014	6	60	25	61	21.3%	0.24 [0.11, 0.55]	8.3%
Pieri 2017	2	46	16	51	13.8%	0.14 [0.03, 0.57]	4.7%
**Subtotal (95% CI)**		**147**		**159**	**51.7%**	**0.22 [0.12, 0.40]**	
Total events	11		55				
Heterogeneity: Tau² = 0.00; Chi² = 0.51, df = 2 (P = 0.77); I² = 0%							
Test for overall effect: Z = 4.89 (P < 0.00001)							
**Total (95% CI)**		**205**		**220**	**100.0%**	**0.39 [0.19, 0.82]**	
Total events	32		84				
Heterogeneity: Tau² = 0.47; Chi² = 14.60, df = 4 (P = 0.006); I² = 73%							
Test for overall effect: Z = 2.51 (P = 0.01)							
Test for subgroup differences: Chi² = 8.22, df = 1 (P = 0.004), I² = 87.8%							
**d. Biological complications (before and after loading) at 8 years of follow-up**							
**d.1. Short implant length: 6 to 8.5 mm**							
Felice 2018	9	60	27	61	100.0%	0.34 [0.17, 0.66]	
**Subtotal (95% CI)**		**60**		**61**	**100.0%**	**0.34 [0.17, 0.66]**	
Total events	9		27				
Heterogeneity: Not applicable							
Test for overall effect: Z = 3.19 (P = 0.001)							
**Total (95% CI)**		**60**		**61**	**100.0%**	**0.34 [0.17, 0.66]**	
Total events	9		27				
Heterogeneity: Not applicable							
Test for overall effect: Z = 3.19 (P = 0.001)							
Test for subgroup differences: Not applicable							
**Total (95% CI)**		**669**		**703**	**100.0%**	**0.37 [0.26, 0.54]**	
Total events	78		229				
Heterogeneity: Tau² = 0.27; Chi² = 32.40, df = 14 (P = 0.004); I² = 57%							
Test for overall effect: Z = 5.15 (P < 0.00001)							
Test for subgroup differences (follow-up): Chi² = 0.84, df = 3 (P = 0.84), I² = 0%							
Test for subgroup differences (short dental implant length): Chi² = 20.34, df = 1 (P < 0.00001), I² = 95.1%							

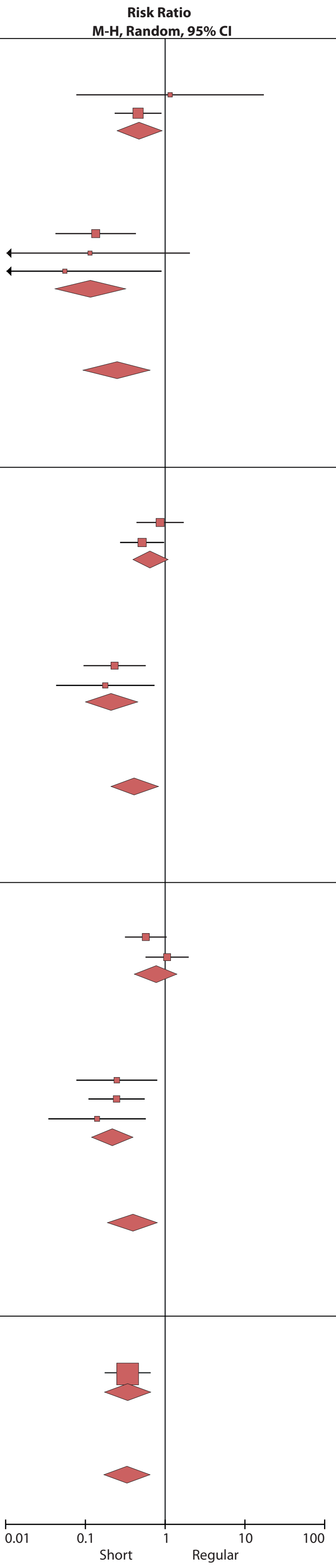

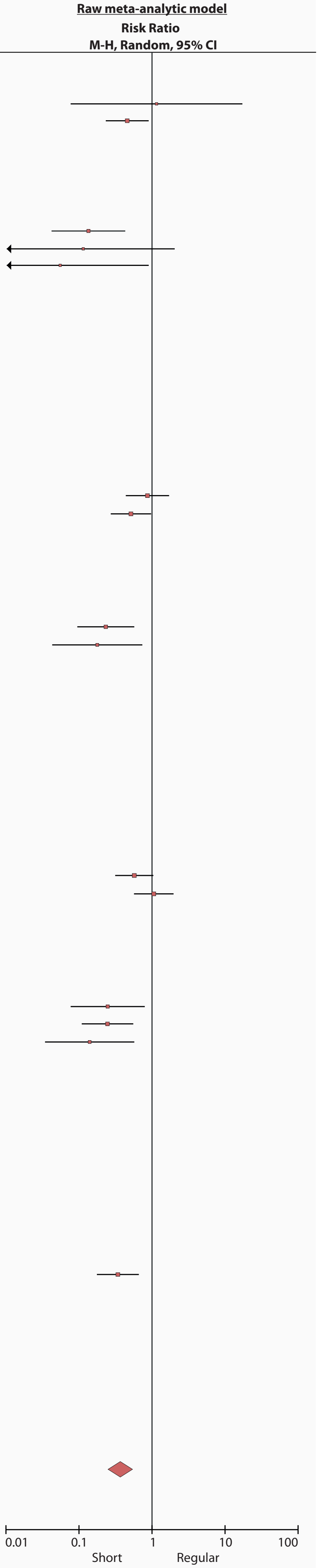

Fig. S3. Forest plot for the random effects meta-analysis assessing the effect of Short implants compared to Regular implants after Bone Augmentation for the posterior athropic mandible on Biological complications. CI: confidence interval, M-H: Mantel-Haenszel, SD: standard deviation

Reference	Short implants Events	Short implants Total	Regular implants Events	Regular implants Total	Weight	Risk Ratio M-H, Random, 95% CI	Raw meta-analytic model Weight
**a. Prosthesis failure and complications at 3 years of follow-up**							
**a.1. Short implant length: 4 to <6 mm**							
Esposito 2014	1	15	0	15	11.2%	3.00 [0.13, 68.26]	2.9%
Gastaldi 2018	1	19	2	20	20.3%	0.53 [0.05, 5.34]	5.4%
**Subtotal (95% CI)**		**34**		**35**	**31.5%**	**0.98 [0.15, 6.28]**	
Total events	2		2				
Heterogeneity: Tau² = 0.00; Chi² = 0.77, df = 1 (P = 0.38); I² = 0%							
Test for overall effect: Z = 0.03 (P = 0.98)							
**a.2. Short implant length: 6 to 8.5 mm**							
Esposito 2011	3	28	4	28	55.4%	0.75 [0.18, 3.05]	14.6%
Felice 2018	0	15	3	14	13.2%	0.13 [0.01, 2.38]	3.5%
**Subtotal (95% CI)**		**43**		**42**	**68.5%**	**0.50 [0.11, 2.18]**	
Total events	3		7				
Heterogeneity: Tau² = 0.24; Chi² = 1.18, df = 1 (P = 0.28); I² = 15%							
Test for overall effect: Z = 0.92 (P = 0.36)							
**Total (95% CI)**		**77**		**77**	**100.0%**	**0.65 [0.23, 1.84]**	
Total events	5		9				
Heterogeneity: Tau² = 0.00; Chi² = 2.17, df = 3 (P = 0.54); I² = 0%							
Test for overall effect: Z = 0.81 (P = 0.42)							
Test for subgroup differences: Chi² = 0.31, df = 1 (P = 0.58), I² = 0%							
**b. Prosthesis failure and complications at 5 years of follow-up**							
**b.1. Short implant length: 4 to <6 mm**							
Esposito 2019	1	17	2	18	9.2%	0.53 [0.05, 5.32]	5.4%
Felice 2019	1	10	0	10	5.1%	3.00 [0.14, 65.90]	3.0%
**Subtotal (95% CI)**		**27**		**28**	**14.3%**	**0.99 [0.16, 6.26]**	
Total events	2		2				
Heterogeneity: Tau² = 0.00; Chi² = 0.78, df = 1 (P = 0.38); I² = 0%							
Test for overall effect: Z = 0.02 (P = 0.99)							
**b.2. Short implant length: 6 to 8.5 mm**							
Felice 2019	1	12	3	12	10.9%	0.33 [0.04, 2.77]	6.4%
Felice 2014	5	27	5	25	39.5%	0.93 [0.30, 2.82]	23.2%
Pieri 2017	5	23	4	22	35.3%	1.20 [0.37, 3.88]	20.7%
**Subtotal (95% CI)**		**62**		**59**	**85.7%**	**0.90 [0.42, 1.92]**	
Total events	11		12				
Heterogeneity: Tau² = 0.00; Chi² = 1.08, df = 2 (P = 0.58); I² = 0%							
Test for overall effect: Z = 0.26 (P = 0.79)							
**Total (95% CI)**		**89**		**87**	**100.0%**	**0.91 [0.45, 1.84]**	
Total events	13		14				
Heterogeneity: Tau² = 0.00; Chi² = 1.86, df = 4 (P = 0.76); I² = 0%							
Test for overall effect: Z = 0.25 (P = 0.80)							
Test for subgroup differences: Chi² = 0.01, df = 1 (P = 0.93), I² = 0%							
**c. Prosthesis failure and complications at 8 years of follow-up**							
**c.1. Short implant length: 6 to 8.5 mm**							
Felice 2018	4	25	3	23	100.0%	1.23 [0.31, 4.90]	15.0%
**Subtotal (95% CI)**		**25**		**23**	**100.0%**	**1.23 [0.31, 4.90]**	
Total events	4		3				
Heterogeneity: Not applicable							
Test for overall effect: Z = 0.29 (P = 0.77)							
**Total (95% CI)**		**25**		**23**	**100.0%**	**1.23 [0.31, 4.90]**	
Total events	4		3				
Heterogeneity: Not applicable							
Test for overall effect: Z = 0.29 (P = 0.77)							
Test for subgroup differences: Not applicable							
**Total (95% CI)**		**191**		**187**	**100.0%**	**0.87 [0.51, 1.49]**	
Total events	22		26				
Heterogeneity: Tau² = 0.00; Chi² = 4.60, df = 9 (P = 0.87); I² = 0%							
Test for overall effect: Z = 0.50 (P = 0.62)							
Test for subgroup differences (follow-up): Chi² = 0.56, df = 2 (P = 0.76), I² = 0%							
Test for subgroup differences (short implant length): Chi² = 0.04, df = 1 (P = 0.85), I² = 0%							

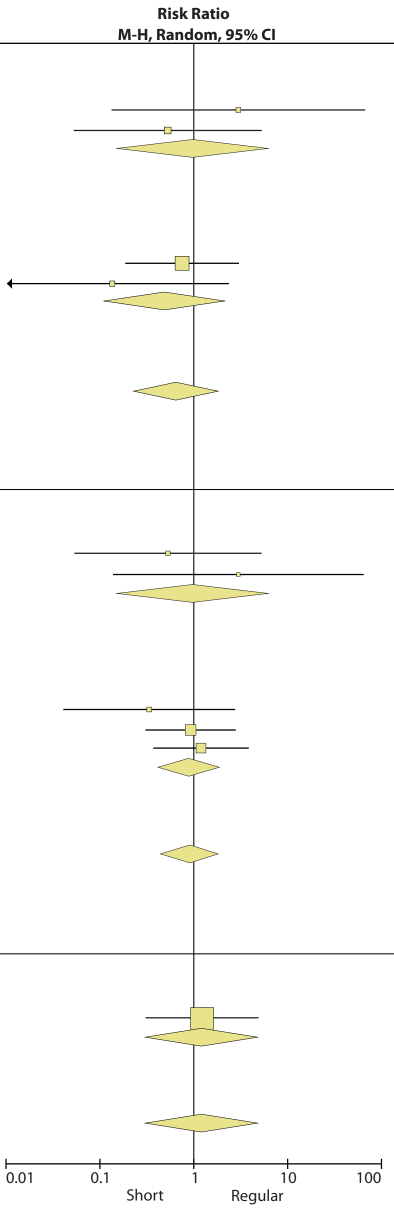

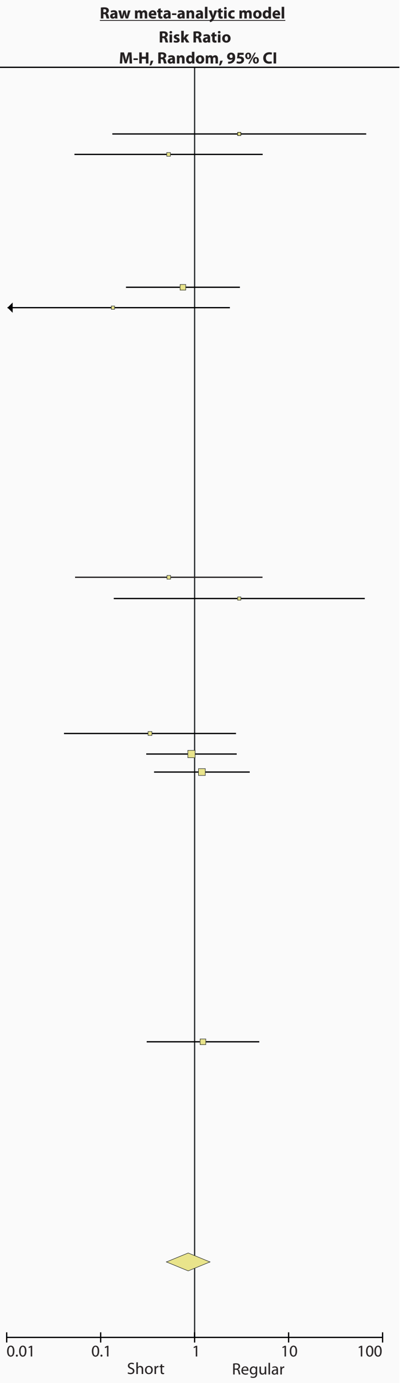

Fig. S4. Forest plot for the random effects meta-analysis assessing the effect of Short implants compared to Regular implants after Bone Augmentation for the posterior athropic mandible on Prosthesis failures and complications. CI: confidence interval, M-H: Mantel-Haenszel, SD: standard deviation

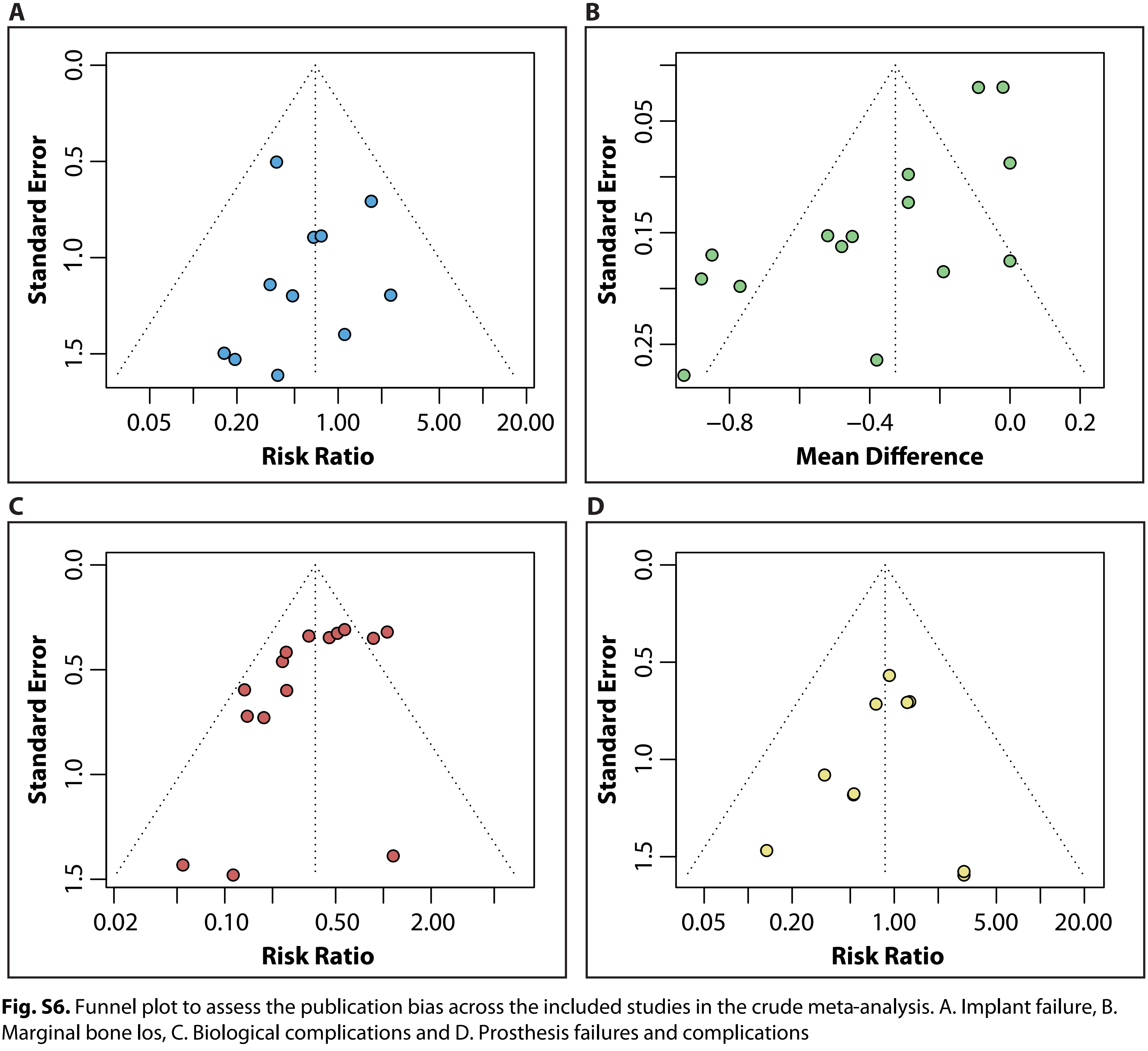

Fig. S6. Funnel plot to assess the publication bias across the included studies in the crude meta-analysis. A. Implant failure, B. Marginal bone los, C. Biological complications and D. Prosthesis failures and complications
